# Coevolution of body size and metabolic rate in vertebrates: a life‐history perspective

**DOI:** 10.1111/brv.12615

**Published:** 2020-06-10

**Authors:** Jan Kozłowski, Marek Konarzewski, Marcin Czarnoleski

**Affiliations:** ^1^ Institute of Environmental Sciences Jagiellonian University Gronostajowa 7, 30‐387 Kraków Poland; ^2^ Institute of Biology University of Białystok Ciołkowskiego 1J, 15‐245, Białystok Poland

**Keywords:** life history, mass scaling of metabolism, negative allometry, hypoallometric scaling, evolution of metabolic rate, evolution of body size, adaptation, physiology

## Abstract

Despite many decades of research, the allometric scaling of metabolic rates (MRs) remains poorly understood. Here, we argue that scaling exponents of these allometries do not themselves mirror one universal law of nature but instead statistically approximate the non‐linearity of the relationship between MR and body mass. This ‘statistical’ view must be replaced with the life‐history perspective that ‘allows’ organisms to evolve myriad different life strategies with distinct physiological features. We posit that the hypoallometric allometry of MRs (mass scaling with an exponent smaller than 1) is an indirect outcome of the selective pressure of ecological mortality on allocation ‘decisions’ that divide resources among growth, reproduction, and the basic metabolic costs of repair and maintenance reflected in the standard or basal metabolic rate (SMR or BMR), which are customarily subjected to allometric analyses. Those ‘decisions’ form a wealth of life‐history variation that can be defined based on the axis dictated by ecological mortality and the axis governed by the efficiency of energy use. We link this variation as well as hypoallometric scaling to the mechanistic determinants of MR, such as metabolically inert component proportions, internal organ relative size and activity, cell size and cell membrane composition, and muscle contributions to dramatic metabolic shifts between the resting and active states. The multitude of mechanisms determining MR leads us to conclude that the quest for a single‐cause explanation of the mass scaling of MRs is futile. We argue that an explanation based on the theory of life‐history evolution is the best way forward.

## I. INTRODUCTION

Enormous diversity of body mass is observed within orders of animals and even narrower clades. This diversification is accompanied by a slower than linear increase of metabolic rates (MRs; see Table 1 for glossary) with body mass, which has fascinated biologists since Rubner (1908), who proposed that the surface‐to‐volume ratio dictates that MR increases with body mass at a rate of 2/3. Subsequently, the 3/4 scaling proposed by Kleiber (1932, 1947) became popular; in this scaling, the exponent was initially not considered a manifestation of biological laws but as an approximation of empirical data rounded to 3/4, which may facilitate utilitarian calculations with a slide rule (Hulbert, 2014). The quest for a unifying explanation of the mass scaling of MRs then began, with heated discussions still continuing regarding whether the exponent is closer to 3/4 or 2/3. This quest has been futile, as illustrated by a recent sequence of papers on basal metabolic rate (BMR) scaling in mammals: White & Seymour (2003) argued for a slope of 2/3, then Savage *et al*. (2004) argued for 3/4, followed by White, Blackburn, & Seymour (2009), Sieg *et al*. (2009) and Capellini, Venditti, & Barton (2010) arguing that neither value was appropriate [see Griebeler & Werner (2016) for review of other papers questioning the universal scaling exponent].

**Table 1:** Glossary of selected terms

Term	Definition
*A*	Resource acquisition rate
BMR	Basal metabolic rate
DEB	Dynamic Energy Budget theory
DEE	Daily energy expenditure, also called the field metabolic rate (FMR) or routine metabolic rate
FAS	Factorial metabolic scope
HDL	Heat Dissipation Limit hypothesis
*m*	Mortality rate
*M*	Maintenance metabolic rate, energy expenditure required for somatic maintenance
MinMR	Minimal metabolic rate; used when distinguishing among BMR/RMR/SMR is not important or is impossible; the lower energy expenditure during hibernation/torpor is not included
MR	Metabolic rate
MTE	Metabolic Theory of Ecology
*P*	Production rate, indicating potential of an organism to produce new tissue (own or offspring)
RMR	Resting metabolic rate
SMR	Standard metabolic rate; ambient temperature should be specified, although in avian studies, often used for the thermoneutral zone with the possibility that the conditions for BMR measurement are not fully satisfied
*w*	Body mass

Recently, White *et al*. (2019) used phylogenetic evidence to show that body mass and MR did not evolve independently but were subjected to correlational selection. Understanding the mechanisms of multivariate selection shaping body mass and MR requires a life‐history approach that considers a relevant fitness measure. Without such an approach, efforts to explain the hypoallometric scaling of MRs with various mechanistic models may be fruitless because body mass is routinely treated as an independent variable, and only MR is perceived as the direct target of selection (see online Supporting information, Appendix S1 for terminology and the form of scaling equations). However, if body mass and MR are considered as coevolving determinants of fitness, then apparent dependent and independent variables are not observed in the relationship between the two traits and selection to maximise fitness could alter either one or both of these traits.

The resulting fitness‐maximizing life history would certainly depend on survival and reproduction, which both require an array of physiological and behavioural compromises at all life stages. To understand such compromises, physiology must be considered through a life‐history lens. Accordingly, physiological and behavioural adaptations do not need to be perfect because the maximization of survivorship or fertility of an individual is not the ‘evolutionary goal’. Deaths of many individuals before reaching maturity can be compensated for by the production of large numbers of offspring by lucky survivors carrying the same genes responsible for a given trait. Thus, there is room for species that produce either large numbers of poorly surviving offspring or small numbers of offspring that survive well, for short‐lived and long‐lived species, for small and large‐bodied species, etc. These alternative life strategies certainly require different metabolic characteristics. Given that evolution takes place in populations, our understanding of the evolution of body size in association with MR requires approaches that focus on populations rather than the functioning of individuals and their homeostasis.

Section II considers the evolution of body size from the perspective of the optimal allocation of resources, and the complex role of MR in this evolution is also discussed. Section III organizes the enormous diversity of possible life strategies around the mutual role of mortality and MR. Section IV, focusing on MR scaling equations, suggests that they only represent statistical relationships between MR and body mass and distinguishes between ‘why’ and ‘how’ questions in explaining the ubiquity of the hypoallometric scaling of MR. The evolutionary ‘why’ question is explored in Section IV.4, and the mechanistic ‘how’ question is investigated in Section V. MRs are sometimes limited by supply, demand, or heat dissipation, although they are usually regulated instead of limited, and the regulation mechanisms are considered in Section VI. Suggested future directions and conclusions are presented in Sections VII and VIII.

## II. BODY SIZE AS AN ADAPTATION

Reproductive value at birth is a universal fitness measure (Charlesworth, 1994) that is equivalent to the expected‐at‐birth number of offspring produced, with future offspring discounted relative to their current value: the later the offspring is placed into the population, the lower the proportion it forms of the future gene pool if the population is expanding and the higher the proportion if a population is shrinking. If the population is stable, as is more or less the case for most vertebrates (Sibly *et al*., 2007), and regulated by fecundity or juvenile mortality/migration, then the reproductive value at birth simplifies to the expected‐at‐birth offspring number. For simplicity, only such ecological scenarios are discussed further; for a discussion of fitness measures under other scenarios, see Dańko *et al*. (2018).

The growth and reproduction of animals are limited by the amount of acquired resources, the quantity or quality of food, or physiological constraints. Animals can also limit food consumption to reduce risky foraging or secure physiological rest necessary for tissue/cell repair (Section VI.3). After basic maintenance costs are covered, those limited resources must be optimally allocated to growth, reproduction and somatic repair to maximize lifetime offspring production. Let us consider first animals that do not grow substantially after maturation (determinate growth). Mechanistically, their final size depends on their birth size, growth rate and growing period length (Kozłowski, 1989). The growth rate depends directly on the difference between the rates at which energy is acquired *A*(*w*) and spent for maintenance *M*(*w*), where *w* is body mass. *M*(*w*) depends on physiological properties and behaviour and is typically a monotonically increasing function of *w*. *A*(*w*) would also monotonically increase under an *ad libitum* food source, although it can take a complex shape depending on food availability for animals of different sizes. Thus, the result of *A*(*w*)–*M*(*w*), called the production rate *P*(*w*), also may adopt complex shapes with regions of concave upward increases (convex, increasing faster than linearly) and concave downwards increases (increasing slower than linearly) and the presence or absence of local maxima (see Appendix S1 for more information on the curvature of functions). Intuitively, the shape of *P*(*w*) is important for the evolution of adult body size because it determines the capacity to produce either new own tissue or offspring. Although somatic growth and reproduction can be carried out simultaneously, resource allocation models predict that at a given time point, all surplus resources not used for maintenance (entire *P*) should be utilized either for growth or for reproduction but not for both processes simultaneously (Kozłowski, 2006). Given a humped shape of *P*(*w*), some researchers envisioned that organisms evolve adult masses that maximize *P* (Sebens, 1987; Reiss, 1989; Brown, Marquet, & Taper, 1993), although this perspective overlooks the populational characteristics of evolution and that the allocation of resources to growth is only an investment in the mortal soma, which is advantageous as long as it increases the expected offspring production (accounting for mortality risk) (Kozłowski, 1992, 2006, 1996*a*). Adult size is not ‘given’ to individuals; rather, it is developed *via* growth. Elongation of a juvenile growth period and thus postponement of reproduction inevitably decreases the likelihood of survival to maturation but allows the body size to increase with the reward of higher reproductive potential. This potential is measured by reproductive allocation and not necessarily by offspring number because larger animals may either produce more offspring or larger, better‐surviving offspring. Life expectancy after maturity, which depends on adult mortality, determines the average time window in which the juvenile investments in the soma are paid back at an adult stage in the form of offspring production. Thus, the expected offspring production, which is a measure of evolutionary competitiveness, must account for adult survivability as well as for the size dependence of the production rate *P*.

**Fig 1:**
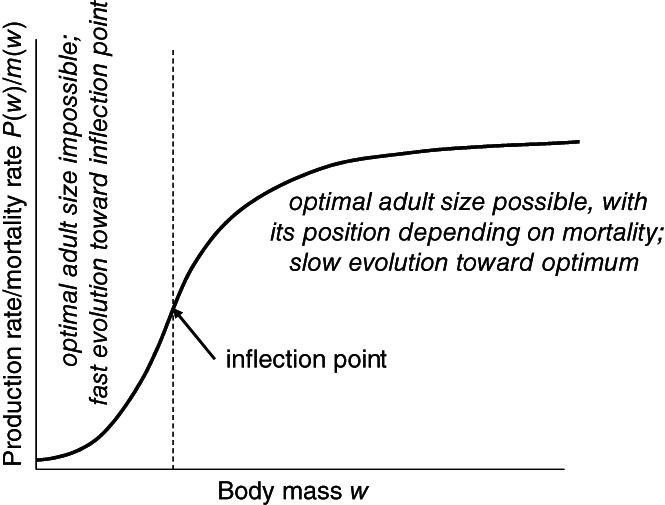
Optimal body size must be placed in the size range for which the production rate divided by mortality rate, *P*(*w*)/*m*(*w*), where *w* is body mass, is concave downwards. *P*(*w*)/*m*(*w*) is expressed in energy units because production is measured in J/day and mortality is measured in 1/day. Because life expectancy is equal to 1/*m*(*w*), this expression measures the expected amount of energy allocated to offspring for an animal maturing at size *w*. If the adult mass is in the range for which the ratio is concave upward, then strong directional selection is expected to increase the body mass rapidly to the point when the shape of this function becomes concave down and then slowly to a size that maximizes fitness. For size‐independent mortality, *m*(*w*) can be removed from the vertical axis legend. See Appendix S1 and Kozłowski (2006) for more details and for explanation of the body size optimization condition: if an increase in body size by 1 J increases the expected offspring production (taking into account mortality) by more than 1 J, then growth is adaptive; otherwise, the use of this energy for reproduction becomes adaptive.

As shown by Kozłowski (1996*c*), the shape of the *P*(*w*)/*m*(*w*) function, where *m*(*w*) is the mortality rate at a given body mass, plays a crucial role in determining an optimal and thus adaptive adult body mass. Because life expectancy for animals that stopped growing and matured at size *w* equals 1/*m*(*w*), the expression *P*(*w*)/*m*(*w*) measures the average expected lifetime amount of energy available for offspring production by animals that survive to maturity. In the simplest case, mortality is independent of body mass. Then, the shape of *P*(*w*) will alone determine the size range in which the optimal adult mass can be placed. Such mass is never placed in the region of *P*(*w*), where the function is concave upward (Fig. 1; Kozłowski, 1996*c*). Certainly, if environmental shifts impose changes in the shape of *P*(*w*), which is driven by factors that include food conditions and thus by *A*(*w*), then body mass can suddenly be displaced to such a region. Because the fitness landscape is flat around an optimal size and steep far away from the optimum (Kozłowski & Uchmański, 1987), strong selection would drive the rapid evolution of body mass towards the region where *P*(*w*) increases at a slower than linear rate with *w* (concave downwards), which would be followed by slower evolutionary changes of body mass under weaker selection until a new optimum is reached. Palaeontologists would probably classify the first stage as a punctuated equilibrium pattern and the second stage as gradual evolution. The new adaptive body mass would be placed somewhere in the concave downwards region of *P*(*w*) but below the local maximum of *P*(*w*) if such a maximum exists. Returns from growth in the form of energy that can be allocated to offspring are diminished with body mass in such regions of *P*(*w*) because of the hypoallometric increase of *P* (Fig. 1). Therefore, the placement of the optimum depends on the mortality rate. Under high mortality, the average return‐of‐investment‐in‐growth period is short; thus, the return must be high, which leads to selection for small size where *P*(*w*) increases rapidly. Under low mortality, longer investments in growth and thus smaller returns per unit of mass increase are compensated by the increased expected future reproduction, which selects for larger adult size. If there is no inflection point, then the outcome is the same except for the absence of the first stage of fast evolution towards the concave downwards range of *P*(*w*). See Appendix S1 for more information on the position of the optimal adult and offspring size.

The evolution of adaptive adult size in relation to production and mortality determines the characteristic distribution of body mass in nature, which is right skewed even on a logarithmic scale (Gaston & Blackburn, 1995; Gardezi & da Silva, 1999; Dixon & Hemptinne, 2001; Knouft & Page, 2003) and even when the mortality rate is size independent (Kozłowski & Gawelczyk, 2002), which corresponds to the dominance of small, but not very small animals. This is because very large animals can evolve only under high production rate and low mortality or modest production and low mortality strongly decreasing with size [if *P*(*w*) has a maximum], very small animals can only evolve under low production rate and high mortality, and animals of moderate size can evolve under either high production and high mortality or low production and low mortality. Thus, we can expect the highest diversity of metabolic strategies and life histories in animals of a moderate size.

The role of size‐independent mortality in shaping optimal body size is often misinterpreted. For example, according to Brown & Sibly (2006, p. 17597), “If death rate is constant, however, fitness depends only on production rate”. This expectation holds only for populations that are almost always in an unconstrained growth phase or regulated by density dependence acting through mortality in an age‐independent way, whereas it does not hold if density dependence acts on the reproduction rate or juvenile mortality/emigration (all three mechanisms affect the number of recruits to the reproducing population), which is likely to occur in vertebrates [Dańko *et al*., 2018 and citations therein]. In such cases, the expected lifetime offspring production is a proper measure of fitness and mortality strongly affects fitness and the adaptive adult mass. According to Brown & Sibly (2006), only strong decreases in mortality or increases in food availability with body mass are expected to drive selection for large size, whereas size‐independent mortality level is neutral to selection on body size. If so, however, the world would be dominated by very small species with extremely short generation times, with rare cases of medium and large species.

The size dependence of mortality is not necessary for the evolution of a broad range of body mass, although it changes this evolution quantitatively by shifting the regions of concave/convex *P*(*w*)/*m*(*w*) left or right relative to such regions in *P*(*w*), and the position of the optimal body mass is then shifted down or up. In terrestrial ecosystems, mortality typically decreases with increasing body mass, which will shift the optimal body mass upwards. If *P*(*w*) has a maximum, then the decrease in the mortality rate with body mass must be extremely high to reach sizes beyond such a maximum (Kozłowski & Gawelczyk, 2002). Such giants with modest mass‐specific MR are rare in nature, and their existence is properly interpreted as an escape from predation pressure.

Seasonality complicates such a simple picture of size evolution because growth becomes optimal following maturity, with a decreasing fraction of resources allocated to growth and an increasing fraction allocated to reproduction year after year (Kozłowski & Uchmański, 1987; Kozłowski, 1996*b*; Czarnoleski & Kozłowski, 1998; Kozłowski & Teriokhin, 1999). Body mass continues to increase towards some asymptotic size, and the difference between the asymptotic and reached‐at‐maturation sizes depends on mortality. Under low mortality, growth after maturation may be negligible, as observed in turtles (e.g. Congdon *et al*., 2012; Omeyer, Godley, & Broderick, 2017), with adult annual survivability occasionally reaching 0.98 (e.g. Chaloupka & Limpus, 2004). The timing of growth and reproduction within a favourable season (growth then reproduction or reproduction then growth) also evolves, and it reflects a compromise between the timing of reproduction that maximizes future prospects of offspring and the timing of allocation activities that maximize reproductive investment (Ejsmond *et al*., 2010). If reproduction late in a season strongly reduces the prospects of offspring, then the winning allocation strategy becomes capital breeding, i.e. storing resources in autumn to fuel early spring reproduction (Ejsmond *et al*., 2015).

**Fig 2:**
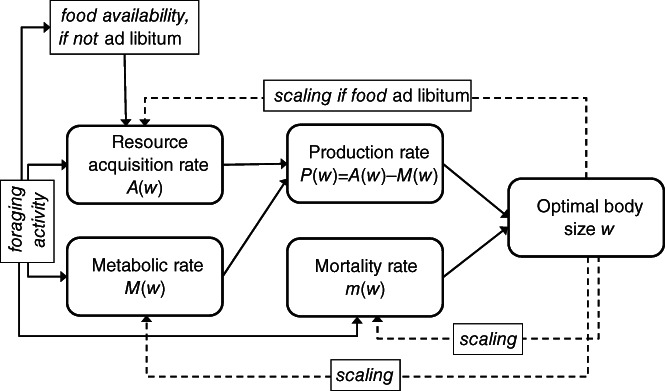
Interrelations between resource acquisition and metabolic and mortality rates in shaping optimal body size.

**Fig 3:**
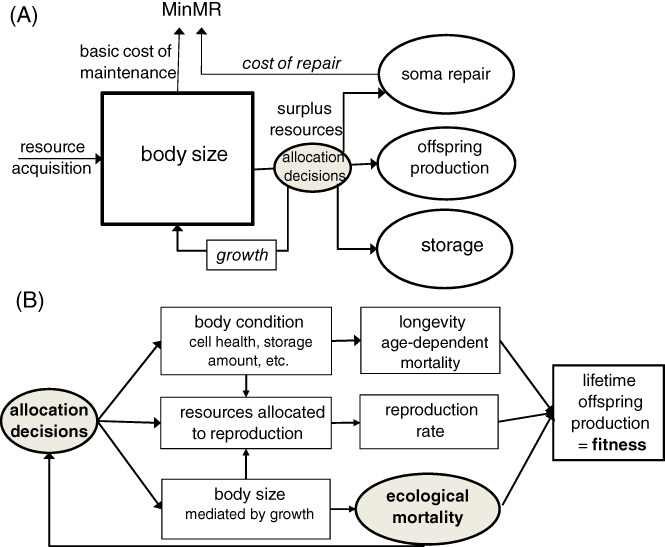
(A) Resource allocation decisions shape life histories *via* sinks for resources. (B) The role of ecological mortality and the consequences of allocation decisions. See also Section VI.3, where we discuss energy‐dependent/independent repair mechanisms. MinMR, minimal metabolic rate.

The role of MR in body mass evolution is manifold (Fig. 2). It is a component of *P*(*w*), with high MR draining resources from tissue production, but sometimes capable of increasing the rate of resource acquisition. The production of new tissues is also never 100% efficient and thus introduces additional metabolic costs. The MR also has a complex effect on mortality, with faster metabolism capable of increasing energetic demands and thus the time allocated to risky foraging, although it may also improve escape from or chasing of prey (for the relationship between MR and behavioural traits, see a recent meta‐analysis by Mathot, Dingemanse, & Nakagawa, 2019) and may be involved in cellular repair processes necessary for long/healthy life, which becomes selectively advantageous under low ecological mortality (Kirkwood, 1990; Cichon, 1997). Allocation decisions (Fig. 3A) that are dependent on resource availability and mortality define the final outcome, fitness, and organismal state variables, such as body size and body condition, and thus translate into longevity and reproductive intensity (Fig. 3B). Section III organizes the enormous diversity of life histories around the mutual role of mortality and MR.

## III. SLOW–FAST/FRUGAL–WASTEFUL CONTINUUM

The concepts of slow–fast (Promislow & Harvey, 1990) and frugal–wasteful (Szarski, 1983) strategies order organisms according to their life history and physiology. Kozłowski, Konarzewski, & Gawelczyk (2003b) surmised that species fall into the plane defined by two axes, with the slow–fast life axis governed by ecological mortality and the frugal–wasteful axis governed by production efficiency. Wasteful organisms are likely built of small cells and have a high MR (Section V.2), and they can grow rapidly if resources are abundant but malfunction under poor conditions. Frugal organisms are likely built of large cells and have a low MR, and they grow slowly even if food is abundant because their ability to process food is constrained, although they can thrive under unfavourable conditions (see Section V.2). Not distinguishing between the axes defined by production efficiency and mortality risk may result in the misinterpretation of correlational or scaling data.

**Fig 4:**
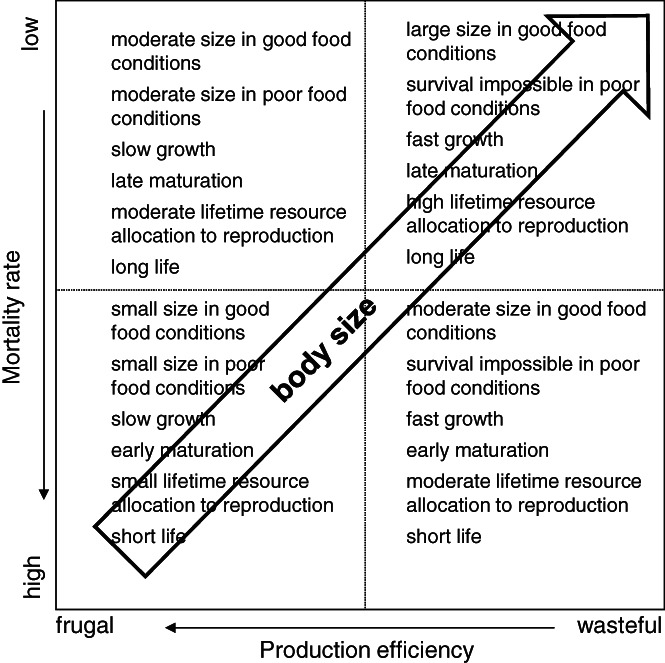
Expected life‐history traits with a predicted trend in adaptive body sizes in relation to production efficiency and ecological mortality risk. Organisms with wasteful physiologies dissipate a larger amount of energy to build a unit of energy into their own or offspring tissues than organisms with frugal physiologies. Under the same physiology, species living under high risk of mortality should mature earlier and have shorter lifespans than species living under a low risk of mortality.

The risk of mortality determines how long organisms thrive, grow, prepare for reproduction and reproduce. Because frugal organisms cannot grow rapidly even when resources are freely available, they will reach only a moderate size in safe environments and a small size in risky environments (Fig. 4). Wasteful organisms can grow to a large size if food is abundant and mortality is low or a moderate size if food is abundant and mortality is high, and such organisms should not be represented in poor‐food environments. Thus, moderate sizes can be found among frugal organisms with a long life expectancy and wasteful organisms with a short life expectancy (Fig. 4). Certainly, the body size of a particular species can be adjusted to local conditions only within its genetic variance and/or phenotypic plasticity. In reality, environments are so diverse that the entire plane in Fig. 4 is filled with species, and their distribution on the plane will affect body mass distributions, the mass scaling of physiological traits, and the scatter of data points around the central tendencies represented by scaling lines.

Although there is no doubt that different characteristics of metabolism (minimal, maximal, field, etc.) are heritable, they are ultimately correlates of fitness components, with selection acting on combinations of metabolic characteristics (Pettersen, Marshall & White, 2018). Using such traits as fitness proxies may lead to misleading interpretations of the direction of selection; thus, their relation with actual fitness measures must be considered (Pettersen, White, & Marshall, 2016; Pettersen, Marshall, & White, 2018). The distribution of species on a plane in Fig. 4 therefore begs the question of whether their MRs, particularly some measures of the minimal metabolic rates (MinMRs), which are the subject of most studies on scaling, may be a target of natural selection. By definition, animals in the MinMR state are not actively involved in reproduction or exposed to environmental conditions that hamper survival. However, selection to minimize MinMR may be directly relevant for sit‐and‐wait strategists, such as pythons (*Python molurus*) (Secor & Diamond, 1997) and endotherms that spend significant amounts of time in the thermoneutral zone, where active foraging is avoided to reduce predation. Weasels (*Mustela nivalis*) are a good example: they kill one prey per day and spend the remaining time safe and warm in the hole of the prey, and in such animals, a higher basal metabolic rate (BMR) would require hunting for more than one prey with a high risk of being killed (Zub *et al*., 2009). However, the associations between MinMR and survival are complex and variable because their nature may vary over time, even within the same species (e.g. the bank vole *Myodes glareolus*; Boratyński & Koteja, 2009). The links between MinMR and reproduction seem more uniformly positive although sex dependent (Boratyński & Koteja, 2010; Boratyński *et al*., 2013; Sadowska, Gebczynski, & Konarzewski, 2013). Overall, the MinMR is most likely a by‐product of the energetic cost of adaptations to a given environment (Clarke & Portner, 2010), and it evolves *via* cross‐links with survival and reproduction (Ricklefs & Wikelski, 2002), which are most likely reflected in the generally positive correlation between MinMR and other types of MRs, particularly daily energy expenditure (DEE) (Auer, Killen, & Rezende, 2017). To understand this complexity, it is necessary to consider the determinants of the MR at the organismal, tissue and cell levels, which are discussed in Sections V and VI. We now consider the status of scaling studies in the context of MR and body mass evolution.

## IV. MASS SCALING OF METABOLISM: WHY SO MUCH BUZZ?

Reviewing mechanistic theories that address the hypoallometric mass scaling of MR is not our aim because many such reviews are available (e.g. Suarez, Darveau, & Childress, 2004; Glazier, 2005, 2014; Kearney & White, 2012; White & Kearney, 2014; Harrison, 2018*a*). Most of these theories ignore the coevolution between MR and body size and the driving force of mortality in this coevolution (but see Harrison, 2017, 2018*a*). The prominent Metabolic Theory of Ecology (Brown *et al*., 2004, hereafter MTE) promises to provide insights into a wide range of ecological processes and patterns, although it suffers from a similar shortcoming. As a starting point, the MTE considers only one determinant of fitness expressed by the expected‐at‐birth offspring number: the dependence of MR on body mass and temperature, either ignoring other fitness determinants such as the rates of resource acquisition and mortality, or including them as correlates of MR. As discussed in Section II, MR, resource acquisition and mortality are mutually interdependent (Fig. 2). Importantly, the model that founded the MTE (West, Brown, & Enquist, 1997) predicts the existence of a universal mass‐scaling exponent of MR (0.75) on the grounds of physics and extremely simplified physiology/anatomy of the distribution network, with an unrealistic fitness measure not rooted in demography. Also, models designed to explain the diversity of the mass‐scaling exponent within the framework of the distribution network limitation do not apply an actual fitness measure (Kolokotrones *et al*., 2010; Newberry, Ennis, & Savage, 2015; Brummer, Savage, & Enquist, 2017).

Despite its weak points (e.g. Agutter & Wheatley, 2004; Kozłowski & Konarzewski, 2004, 2005; O'Connor *et al*., 2007; Apol, Etienne, & Olff, 2008; del Rio, 2008; Agutter & Tuszynski, 2011; Glazier, 2015; Clarke, 2017), the MTE revived interests in the mass scaling of MR. However, it became evident that the value of the mass‐scaling exponent for MR is not invariable and universal, which is contrary to the firm predictions of the MTE, and it differs among taxa (Hayssen & Lacy, 1985; Bokma, 2004; Clarke, Rothery, & Isaac, 2010; Isaac & Carbone, 2010; White, Frappell, & Chown, 2012; Uyeda *et al*., 2017), depends on temperature (White *et al*., 2008; Clarke *et al*., 2010), season (Vézina *et al*., 2012), energy expenditure (Glazier, 2008; White *et al*., 2008), mass range (White & Seymour, 2005), climate (Lovegrove, 2000, 2003; Rezende, Bozinovic, & Garland, 2004) and can change with ontogeny (Glazier, 2005, 2006). The unfruitful quest for a single scaling exponent and a single cause of hypoallometric scaling was perfectly characterized by Suarez *et al*. (2004, p. 533) as “Single‐cause explanations vs. how animals work”. Perhaps the purpose of scaling studies should be questioned. Has any crucial biological problem(s) been solved with so much effort? If the answer is ‘yes’, then advocates should clearly formulate the problems to make them testable. However, if the answer is ‘no’, then an open discussion is badly needed to highlight fruitful directions for future work. Without such a discussion, scaling research may flourish without any scientific progress because expanding databases and easy‐to‐use numerical tools make this kind of work relatively easy. To open such a discussion, it is necessary to study first the anatomy of the scaling approach, which we address next.

### (1) Scaling equation: biological law or approximation of non‐linearity?

Emerging data show that the log transformation of MRs and body mass (see Appendix S1) does not always entirely remove non‐linearity in interspecific comparisons (Hayssen & Lacy, 1985; Dodds, Rothman, & Weitz, 2001; Kozłowski & Konarzewski, 2005; Painter, 2005; Packard & Birchard, 2008; Clarke *et al*., 2010; Kolokotrones *et al*., 2010; Ehnes, Rall, & Brose, 2011; White & Kearney, 2014; Griebeler & Werner, 2016) or intraspecific comparisons (Glazier, 2005; Moran & Wells, 2007: Czarnoleski *et al*., 2008; Seymour *et al*., 2013; Starostova *et al*., 2013). More data and more sophisticated analyses will likely yield new such cases. If allometric functions are treated as biological laws instead of as useful approximations, then the effort of future research may be invested in unfruitful explanations of such curvilinearity. A more realistic approach is to treat allometric functions as reasonable but imperfect descriptions of general (but not all) trends in data. Allometric functions are indeed highly flexible, although they have serious drawbacks: (*i*) they are always concave upward in hypermetric allometry (also called positive or superlinear; with a scaling exponent greater than 1) or concave downwards in hypometric allometry (also called negative or sublinear; with a scaling exponent lower than 1) across the wide range of body mass, and (*ii*) the scaling exponent must remain constant across the wide range of body mass. Thus, the allometric approximation is poor if there is an inflection point in a relationship or if small and large organisms exhibit different scaling. If the scaling is shallow for small animals, steep for medium‐sized animals and shallow for large animals (or the reverse), a function with an inflection point would approximate this relationship considerably better than the current allometric approximations. Fitting segmented linear regressions on a log–log scale can be a convenient technical solution in this case, as well as in cases when the regression slopes change without inflection point(s). Considering such changes in regression slopes is especially important in intraspecific mass scaling (see Glazier, 2005).

### (2) Mass scaling of MR: satisfied by a general trend or surprised by the residual variance?

Here, the answer depends on the purpose. For researchers interested in the rate of energy flow through a taxon inhabiting a given habitat, predicting the average rate would suffice. Considering that the intraspecific variance of body mass is typically ignored in such calculations and that a large error in the estimation of population density is unavoidable, the bias introduced by ignoring deviations of species from the general trend is negligible. In studying the evolution of MRs, the residual variation cannot be ignored because it reflects deep differences in the biological properties of species and occasionally of individuals. In fact, the log–log scale is often misleading and hides information, e.g. that two species with the same mean body mass differ in MRs by more than an order of magnitude (Hayssen & Lacy, 1985; Careau *et al*., 2009). There is also substantial intraspecific MR variation that is not explained by intraspecific mass scaling (Speakman, Krol, & Johnson, 2004a; Biro & Stamps, 2010; Burton *et al*., 2011; Konarzewski & Ksiazek, 2013; White & Kearney, 2013). Differences in animal personality mean that body size may explain only a small part of the variation in within‐species relationships (Careau *et al*., 2008; Halsey *et al*., 2019), and individual differences in MR may constrain behaviour (Biro & Stamps, 2010; Biro *et al*., 2018).

If a regression function is used to predict the value of one variable, for example, MR from body mass, then scaling relationships based on a central tendency are sufficient and the mechanisms shaping the allometries do not need to be invoked because the equations represent only a statistical model. By contrast, mechanistic models must be rigorous in both assumptions and reasoning, and must not only explain the central tendencies but also examine the causes of residual variation. Clarke (2004, 2006) discusses the difference between these two classes of models and argues that the MTE actually represents a set of statistical models rather than mechanistic models based on first principles. We agree with this and advocate treating scaling equations as not always perfect approximations of non‐linearity. Accepting such a view frees us from studying in detail mathematically complex models, which are often produced by biologically oriented physicists and based on fundamental physical principles while ignoring the role of evolution in producing complex and diverse metabolic patterns (e.g. Santillán, 2003; Demetrius, 2006). Certainly, scaling equations prove to be a useful statistical tool in addressing deeper phenomena, e.g. by removing mass dependence in the search for the postulated negative relationship between production rates and mortality.

### (3) Interspecific scaling ≠ intraspecific scaling

**Fig 5:**
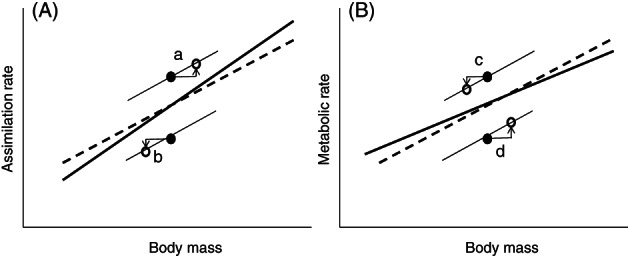
Schematic explanation of why interspecific scaling should be steeper for assimilation (A) and shallower for metabolic rate (B) than intraspecific scaling. Intraspecific relationships for both species are assumed to be the same for the metabolic rate in A and for the assimilation rate in B, which means that the species with the higher assimilation rate have a higher production in A, while the species with the higher MR have a lower production in B. Dashed lines represent average intraspecific relationships, thin lines represent species‐specific relationships, and thick lines represent the resulting interspecific relationships. Filled circles represent the body size of species before body size optimization, and open circles represent body size after optimization. Because the filled circles lie one above another, their departure from the average is neutral with respect to the interspecific slope. Species a and b have the same parameters that describe the size dependence of metabolic and mortality rates, while species a has a higher rate of resource assimilation. The production rate of species a as well as its optimal size at maturity will be higher. Thus, the data point for species a on a log body size–log assimilation rate plane will be placed higher than that for species b and to the right, whereas species b will be shifted to the left, which reduces the variance caused by the higher/lower assimilation (A). For the assimilation rate, the interspecific slope will be steeper than the average intraspecific slope because the interspecific line is pulled upwards on the right and downwards on the left. If species c and d have the same assimilation and mortality rates but species c has a higher MR than species d, then the production rate of c will be lower than that of d, which will also affect the optimal body size (B), and the interspecific slope will be lower than the average intraspecific slope.

For a given species, MRs at different activity levels, BMR, DEE or maximum metabolic rate (MMR), have been shaped during a long evolutionary history. Section II indicated that the same history is also involved in the evolution of an adult size according to the effects of MR on offspring production and mortality. Thus, the within‐species mass scaling of adult MRs and not the interspecific scaling affects the evolution of adult size in a given species. For a group of different species, the evolved body sizes with their accompanying MRs mechanistically and statistically produce the interspecific scaling of MRs. Unfortunately, models aimed at explaining scaling exponents notoriously mix intraspecific and interspecific levels by assuming implicitly that the scaling and its relevance to evolutionary processes are identical on both levels. This implicit assumption, which is by no means granted, results from considering body mass as an independent variable rather than a trait evolving in concert with metabolic levels as indicated by White *et al*. (2019). As shown by Kozłowski & Weiner (1997) *via* life‐history modelling, the coevolution of body size and MR may cause the interspecific mass scaling of MR to be shallower than an average intraspecific scaling (Fig. 5, see also Appendix S2).

The intraspecific scaling of MR among adults requires more attention. Certainly, it is not an ontogenetic allometry because growth and reproduction may require different physiologies and thus create different metabolic patterns. Let us first consider animals that do not grow substantially after maturity. Conceptually, we should imagine an evolving lineage with different adult body sizes and corresponding MRs. In practice, we can exploit the within‐species variance of adult body size but remember that only a part of this phenotypic variance reflects genetic variance, e.g. some small animals may represent a group with unlucky individual histories and should be excluded from calculations. The situation is easier for species that grow intensively after maturity, such as most fish, amphibians or reptiles. Their adult size is represented by the size at maturity and the asymptotic size. Because they grow and reproduce each year, their MRs should be measured in the same phase across all sizes. If the mass scaling of production rate is studied, which seems more justified from an evolutionary point of view than studying MRs (see Section II), then determining the yearly reproductive output for individuals of different sizes is insufficient because larger individuals devote longer parts of the season to reproduction and shorter periods to growth (Kozłowski, 1996*b*; Czarnoleski & Kozłowski, 1998); thus, yearly reproductive output increases with mass faster than physiological capacity to produce offspring tissues (Lester, Shuter, & Abrams, 2004; Barneche *et al*., 2018; Marshall & White, 2018).

### (4) Hypoallometric scaling of MR: the question of ‘why?’

Even if we treat scaling equations as a statistical description and accept the diversity of scaling exponents (Section IV.1), the ubiquity of hypoallometric MR scaling requires two types of explanations: ‘how?’ and ‘why?’. The answer to ‘how’ refers to mechanistic grounds and is addressed in Section V. The answer to ‘why’ is less straightforward and more hypothetical. Apparently, small species with high‐on‐average mass‐specific MRs are equally successful in passing genes to future generations as large animals with low‐on‐average mass‐specific MRs because they coexist; therefore, a lower MR cannot be considered a disadvantage (Brown, Hall, & Sibly, 2018), which is at least implicitly suggested by the models addressing supply limitations. Following Section II, adult size evolves to its adaptive value that must be placed in the region of *P*(*w*)/*m*(*w*) (and likely also of *P*(*w*)) when the function is concave downwards. Unfortunately, hundreds of papers are devoted to MR scaling, whereas we know little of *P*(*w*) and its relation to MR. Sibly & Brown (2007) analysed interspecific scaling of *P*(*w*) in mammals, calculated on the basis of offspring mass production. Mass‐specific *P*(*w*) decreases with body mass, and the exponents differ among clades but roughly resemble the exponents for mass‐specific BMR. Similarly, Peters (1983) documented similarity in scaling exponents for production and respiration. If *P*(*w*) in adult life is more or less proportional to BMR, then the intraspecific scaling of both should be hypoallometric. Note that this does not mean automatically that MR should also scale hypoallometrically at an interspecific level. As shown by Kozłowski & Weiner (1997), such hypoallometric within‐species scaling of MR translates *via* coevolution with body mass to a hypoallometric interspecific scaling of MR, albeit shallower than the average intraspecific scaling (Section IV.3). Because particular species occupy different places on the frugal–wasteful continuum (Section III), data points are scattered around the regression line on the log–log scale. Remembering that small body mass evolves in response to high mortality and/or shallow mass dependence of production rate (Section II), the model based on MR–body mass coevolution explains not only the ‘why’ aspect of hypoallometric interspecific scaling if production rate and MR are correlated but also the existence of broad scattering of species‐specific data. However, it does not answer the ‘how’ question for the hypoallometric scaling of MR.

## V. MECHANISTIC EXPLANATION OF HYPOALLOMETRIC MR SCALING

### (1) Body composition and metabolic activity of tissues

What mechanism may account for a decline in the mass‐specific MRs during evolutionary increases in body mass? There are two non‐exclusive options: the fraction of energetically costly organs/tissues decreases or the mass‐specific MR of body components decreases.

Let us distinguish between metabolically inert organs, such as the skeleton, body fluids, hair, feathers and fat, and metabolically active organs, such as the heart, liver, intestine, kidney or brain, and muscles, which have a relatively low MR at rest and a very high rate at full work. The question is how the participation of these organs changes with body mass and lifestyle. This question must be answered with fitness maximization in mind. For instance, the amount of skeletal material in a body is usually viewed entirely from an engineering perspective, which predicts a faster‐than‐linear increase in skeletal mass with body mass (Schmidt‐Nielsen, 1984; Alexander, 1997). A life‐history perspective suggests other scenarios: a delicate skeleton produces a fragile body but provides more surplus energy for growth or reproduction because less muscle work is required for motion, and the accidental death of some individuals may be over‐compensated by enhanced reproduction of lucky survivors. In fact, bone fracture seems common among primates but is not necessarily a death sentence. Bulstrode (1990) showed that 12–34% of museum specimens had signs of healed bone fracture, which most commonly occurred in young animals.

If the amount of less‐active body parts increases disproportionately in larger organisms, then the scaling of MinMR becomes hypoallometric. Phylogeny‐informed analyses of mammals show that after excluding elephants (which yield a curved relationship on a log–log scale), the scaling exponent for skeleton is 1.02 (White & Kearney, 2014), which is not distinguishable from isometry. In mammals, blood mass scales isometrically with body mass (Peters, 1983; Prothero, 2015). In birds, the mass‐scaling exponent for fat is 0.94 (Daan, Masman, & Groenewold, 1990) or 0.92 (Gavrilov, 2014). In a data set for 100 mammalian species, a phylogenetically informed analysis revealed a proportional increase in adipose deposits with body mass (A. Antoł & J. Kozłowski, in preparation). Thus, the proportion of metabolically inert organs cannot be responsible for hypoallometric scaling, at least in birds and mammals. The same conclusion was achieved by Li *et al*. (2016) for cyprinid fish.

Metabolically active organs use a disproportionately high amount of energy. In humans, the MRs of the brain, liver, kidneys and heart together account at least for 59% of resting metabolism, although these organs constitute less than 6% of body mass (Gallagher *et al*., 1998; Javed *et al*., 2010). The MR of nervous tissue is high even at rest because of the need to maintain the membrane electrochemical potential (Kuzawa *et al*., 2014) and other processes that are not fully understood but are independent of external stimuli (Raichle, 2006). The costs of the brain constitute 20% of the DEE in 15‐year‐old adolescents and 30% of the DEE and more than 60% of the resting metabolic rate (RMR) in 5‐year‐old children (Kuzawa *et al*., 2014). In different strains of laboratory mice that differed in BMR by 30%, the mass of the liver, intestine, kidney and heart constituted from 14.3 to 19.4% (16.6% on average) of the body mass, although the estimated indirect metabolic cost of these organs was approximately 50% of the BMR; interestingly, differences in masses of the internal organs explained 52% of between‐strain and within‐strain differences in the BMR (Konarzewski & Diamond, 1995). Artificial selection for either a higher or lower BMR in mice resulted in the evolution of a 40% difference in the mass‐specific BMR, which was associated with alterations in the mass of the heart, liver, small intestines and kidneys, food consumption, milk production, voluntary activity, core body temperature, cell membrane composition, cell size and other traits relevant to whole‐body metabolism (e.g. Książek, Czerniecki, & Konarzewski, 2009; Brzek *et al*., 2014; Maciak *et al*., 2014; Sadowska *et al*., 2015b). Because artificial selection is based almost exclusively on existing variation (Sadowska *et al*., 2015a), its success shows the potential for change after possible shifts of selection in nature.

A phylogenetically informed re‐analysis of Daan *et al*. (1990) data on 22 bird species revealed isometric interspecific scaling exponents for kidney, heart and liver masses of 0.99, 0.97 and 1.02, respectively, and a negligible phylogenetic signal (A. Antoł & J. Kozłowski, in preparation). Brain mass scaled sub‐linearly with a slope of 0.71, and it had a very strong phylogenetic signal. A phylogenetically informed analysis of a data set of 100 mammalian species revealed the following mass‐scaling slopes for organ mass: 0.70 (brain), 0.84 (kidneys), 0.89 (liver), 0.92 (heart), 0.92 (digestive tract) and 1.00 (lungs), with hypoallometric scaling of the first three organs (A. Antoł & J. Kozłowski, in preparation). The phylogenetic signal was strong for the brain and digestive tract, weak in the kidney, heart and lung, and negligible in the liver. These results show clearly that energetically demanding organs, such as kidney, heart and liver, scale closely to isometry in birds and have no effect on whole‐body scaling. The hypoallometric scaling of the kidney, liver and heart in mammals is too steep to explain fully the hypoallometric scaling of the whole‐body MR. Thus mass‐specific MR, rather than relative mass of these organs must decrease with body mass. However, almost no data are available on the MRs of other tissues/organs than brain and muscles in mammals [but see data in Wang *et al*., 2001 and Porter, 2001].

High energetic demand of the brain in birds and mammals combined with the shallow mass scaling of brain mass may contribute substantially to the hypoallometric scaling of MR. In many studies aimed at investigating the effect of brain size on MR, the effect of body mass is removed for both MR and brain size. After such treatments, the correlation between the residual brain mass and the residual MR may only explain, usually partially, the scatter of data points around the log body mass–log MR regression line and cannot explain the contribution of the brain to the hypoallometric scaling of MR (Harrison, 2018*b*). The same applies to other energy‐demanding organs: scaling shallower than isometry contributes to the hypoallometric scaling of MR, even if the correlation disappears after the effect of body mass is removed.

Selection for larger‐than‐average brain size (higher encephalization) increases fitness through enhanced survivability among other factors (e.g. Sol *et al*., 2007). An increase in relative brain size requires additional resources, which can be acquired *via* a change in diet or digestive capacity that often increases with body mass (Navarrete, van Schaik, & Isler, 2011) or *via* spared expenditures from other functions, such as growth and reproduction, which becomes beneficial if a larger brain increases survival as discovered in birds (Sol *et al*., 2007) and some primates (Allman, McLaughlin, & Hakeem, 1993). Therefore, a relationship between the relative brain size and MinMR may be sensitive to the biological characteristics of studied taxa. An effect of relative brain size on the MinMR was found by Dworak *et al*. (2010) in 51 placental mammals, by Isler & van Schaik (2006) in 347 mammals, by Weisbecker & Goswami (2010) and Genoud, Isler, & Martin (2018) in placental but not marsupial mammals, and by Sobrero *et al*. (2011) in rodents. In carnivores, the relationship was found by Genoud *et al*. (2018) but not by Finarelli (2010).

The effects of muscle mass are important because of the huge difference in the muscle MR between rest and work, which translates to the difference between MinMR and MMR. Resting muscles are not particularly expensive per unit mass and only account for 2.29 kJ/kg/h in humans compared to those of the heart and kidneys (77 kJ/kg/h), the brain (542 kJ/kg/h), or the liver (35 kJ/kg/h) (Gallagher *et al*., 1998). Nevertheless, even at rest, an organism devotes large amounts of energy to muscles because of their considerable proportion of body mass, approximately 40% (Gallagher *et al*., 1998) or 45% (Egginton, 2009) in humans. Muscle mass scales isometrically with body mass in mammals (Raichlen *et al*., 2010; Muchlinski, Snodgrass, & Terranova, 2012; Prothero, 2015) and birds (Daan *et al*., 1990). Hence, the existence of athletic and less‐athletic animals (Weibel *et al*., 2004; Zhang *et al*., 2014) may only explain a part of the data scattering around the log body mass–log MinMR/MMR regression lines, not the hypoallometric scaling of these relationships.

At rest, the heart works slowly because resting muscles require less oxygen and resources. The same heart supplies blood to muscles during escape or pursuit when the MR may increase many‐fold (Weibel & Hoppeler, 2005) and the work of muscles accounts for 90% of the energy consumption of an organism (Taylor, 1987). The factorial metabolic scope (FAS), i.e. the ratio of the MMR dictated mostly by muscle mass to the MinMR with muscles at rest, varies among taxa. In birds and mammals, the FAS slowly increases with body mass, with a scaling exponent of 0.15 (Bishop, 1999). In three species of marine fish, this ratio is 1.5 early in life and 2–4 in the later developmental stages (Killen *et al*., 2007). Overall in adult teleost fish, FAS varies greatly from 1.80 to 12.36 (Killen *et al*., 2016). Pelagic species have access to a good supply of oxygen and food and are athletic, having high protein content in muscles and high RMR and MMR. Conversely, benthic species exposed to low oxygen and food availability, are sluggish and have low RMR and MMR (Killen *et al*., 2016). Thus, teleost fish provide an excellent example of the wasteful and frugal strategies described in Section III.

The relative size of metabolically active organs, especially of the brain, affects the MR towards hypoallometric scaling but is unlikely to explain this pattern fully. Thus, the decrease of the mass‐specific MR of active organs with body mass must also play a role. According to Wang *et al*. (2001), the mass‐specific MRs of organs decreased with body mass in the BMR state for five mammalian species (rat, rabbit, cat, dog and human), most rapidly in the liver (exponent − 0.27), moderately in the brain and heart (−0.12), and most slowly in the kidneys (−0.08); the exponent for the remainder of the body was −0.17. In nine species of mammals, the mass‐specific MR of hepatocytes decreased with body mass with the exponent − 0.18 (Porter, 2001). Karbowski (2007) reported an exponent of −0.14 for glucose metabolism in the brains of 10 mammals ranging in size from mouse to human. According to these data, the total BMR in mammals must be hypoallometric as the proportion and activity of organs with high energy demands decreases with body mass. Unfortunately, the analyses of Wang *et al*. (2001) and Karbowski (2007) are not phylogenetically informed and are based on a small number of species. Clearly, more data are needed to estimate the quantitative role of the decrease in size of energy‐demanding organs and their mass‐specific MRs with body mass in shaping the hypoallometric scaling of MR in different classes of vertebrates.

Because the relative sizes and MRs of organs are likely to differ among species, populations or even individuals (Careau *et al*., 2008) and are strongly mass dependent, body composition should not be ignored when studying intraspecific or interspecific scaling. We are not the first to draw attention to this phenomenon [see reviews by Suarez & Darveau, 2005 and Suarez *et al*., 2004 who invoke the revolutionary paper by Krebs, 1950; see also Painter, 2005 and Glazier, 2014, 2018a for a historical survey of this issue since the early 20th century]. However, most modern theories aimed at explaining the scaling of MR ignore this unavoidable dependence of MR on body composition (but see Harrison, 2017).

Our approach to the roles of metabolically active and inert body components in the mass scaling of metabolism is at odds with the approach represented in the Dynamic Energy Budget (DEB) theory (Kooijman, 2010), which ignores the life‐history perspective (for details see White & Kearney, 2013). The DEB uses the term ‘structure’ for metabolically active components and ‘reserve’ for inert body components, stressing that these components are concepts rather than measurable traits. Here, we focus on body part masses that can be directly measured. In the DEB theory, body size is treated as an emergent property of metabolism (Lika, Augustine, & Kooijman, 2018) and not derived from physiological mechanisms together with allocation ‘decisions’ dependent on mortality, as in the approach considered here (see Section II). Below, we link these allocation ‘decisions’ not only to changes in body components but to their underlying cellular architecture, which is another important property largely ignored in scaling studies.

### (2) Body mass, cell size and MR

Unlike unicells or eutelic multicellular organisms with a fixed number of cells, the body sizes of non‐eutelic animals can evolve *via* alterations in cell size as well as cell number. Unfortunately, the cellular basis of body size evolution has rarely been studied (Javed *et al*., 2010), although it can help address other factors that shape metabolic scaling (Fig. 4).

All else being equal, achieving a larger adult size requires either prolonged growth or faster growth at a juvenile stage. The selective advantage of one or the other solution depends on food availability, external mortality and trade‐offs: (*i*) fast growth may be more expensive/less efficient; (*ii*) fast growth requires a high supply of building materials, which requires intense foraging that may be dangerous; and (*iii*) rapidly building new tissue may compromise quality control, resulting in accelerated senescence. If food is abundant, then trade‐off (*i*) may be less important. Optimality under trade‐offs (*ii*) and (*iii*) depends on ecological mortality. If ecological mortality is high, then the trade‐off between the growth rate and longevity may not affect fitness. Ultimately, any growth strategy that evolves requires specific structural and biochemical adaptations that will likely affect the MinMR. However, the evolution of growth rates and cell sizes should not be considered separately: animals with large cells have low rates of embryonic development and grow slowly (Raichlen *et al*., 2010; Muchlinski *et al*., 2012). In fact, growth rate, cell size, cell number and cellular metabolism are jointly regulated by common signalling pathways, such as the TOR (target of rapamycin) and Hippo‐YAP (yes‐associated protein) pathways (Guertin *et al*., 2006; Csibi & Blenis, 2012). The genes that control these pathways in flies are differentiated along latitudinal clines in conjunction with cell size and body size (De Jong & Bochdanovits, 2003). The activities of such pathways also explain coordinated cell size changes in different tissues during evolutionary differentiation of species of mammals, birds and amphibians (Kozłowski *et al*., 2010; Czarnoleski *et al*., 2018). A ubiquitous correlation between nucleus size and cell size, which is visible at intraspecific (Maciak *et al*., 2014) and interspecific levels (Kozłowski *et al*., 2010), suggests the involvement of a cytological mechanism in cell size regulation. The rescaling of cells may also involve changes in the amount of DNA (the so‐called C‐value), which is associated with polyploidy (Otto, 2007), the activity of transposons (Sun *et al*., 2011; Ji & DeWoody, 2016) and other mechanisms that produce repeated sequences (Gregory, 2001). In the evolution of a lineage, an indel (insertion–deletion) process may be biased, thereby increasing or decreasing the C‐value (Hessen, 2015). If cell size affects fitness, then indel processes are under selective control and non‐coding DNA may not be a non‐adaptive effect of drift; therefore, the C‐value enigma, which is the lack of a close relationship between the DNA amount and organismal complexity (Gregory, 2001), may result from different selection pressures on metabolism and growth rates (Hessen *et al*., 2010; Hessen, Daufresne, & Leinaas, 2013; Hessen, 2015).

Studies on the relationship between cell size and MR are limited, which is likely related to Rubner's view that no such relationship exists (after Ellenby, 1953; Rubner, 1908). Motivated by Rubner's work, Ellenby (1953) compared the MRs of diploid and triploid *Drosophila melanogaster*. After failing to find such a difference, he concluded that “In view of these findings, the extensive investigation of cell size was hardly justified” (Ellenby, 1953, p. 482). Ellenby's conclusion may have been premature considering the low statistical power of his study and the fact that most data points for triploids lie below the regression line. We now know that cells require substantial amounts of ATP for ion transport across the plasmalemma to maintain the electrochemical potentials that keep cells alive, and these costs can constitute 20–30% of the energy budget of cells (Rolfe & Brown, 1997; Wu *et al*., 2001). With increasing cell size, the cell surface area/volume ratio decreases and a smaller fraction of metabolism is needed for ion transport (Davison, 1955; Szarski, 1983; Kozłowski, Konarzewski, & Gawelczyk, 2003a). This hypothesis was recently directly supported by the finding that larger fibres of skeletal muscles in marine crustaceans and fishes are less metabolically expensive to maintain and the cost of maintaining the membrane potential is proportional to the fibre surface‐to‐volume ratio (Jimenez, Dillaman, & Kinsey, 2013). Thus, bodies built of larger cells should be more economical (frugal; Szarski, 1983). Indeed, animal species with low mass‐specific MRs tend to have large cells. The erythrocyte volume‐specific MR in amphibians is negatively correlated with erythrocyte size (Goniakowska, 1970). The size of erythrocytes is negatively correlated with the mass‐corrected RMR in birds (Guertin *et al*., 2006; Csibi & Blenis, 2012; Czarnoleski *et al*., 2018), mammals (Vinogradov, 1995), eublepharid geckos (Kozłowski *et al*., 2010) and amphibians (Gregory, 2003). In loaches (*Cobitis* spp.), triploid fishes have larger erythrocytes and lower mass‐specific metabolism than diploids (Maciak *et al*., 2011). According to Darveau *et al*. (2002), the energy demand of the Na^+^ pump scales with body mass with a coefficient of 0.72 under BMR conditions, which may partially result from larger animals having larger cells on average. In 121 species of mammals, phylogenetically informed analyses showed a positive correlation between genome size, which is a proxy for cell size, and body mass, albeit with a broad scattering of data that is partly explained by the very strong phylogenetic signal (Pagel's *λ* = 0.91) (Tang *et al*., 2020).

Cell size should affect the MinMR at a given body mass, and the mass scaling of cell size should affect the mass scaling of the MinMR; however, as discussed in Section III, the MinMR is rarely a direct target of selection. Although large cells are less expensive to maintain, their relatively small cell membrane area can slow the supply of oxygen and nutrients, creating a ceiling for the MR, which may be of importance under ecologically relevant metabolic states. Excessive crowding of molecules in highly active cells may also limit the rate of some reactions and thus of the MR, especially MMR, through disturbed diffusion (Mittal, Chowhan, & Singh, 2015; Fernandez‐de‐Cossio‐Diaz & Vazquez, 2018), with a possible solution that cell size in such tissues is positively related to MR or even changes dynamically with the current activity of the tissue. For example, in mice selected for high and low BMR, erythrocytes and skin epithelium cells were smaller in high‐BMR mice, whereas cells in highly active organs, such as hepatocytes, kidney proximal tubule cells and duodenum enterocytes, were larger than those in other lines (Maciak *et al*., 2014). A similar mechanism was also invoked to explain the intraspecific patterns in thermal‐plasticity of different cell types in terrestrial snails (Czarnoleski, Labecka, & Kozłowski, 2016) and Madagascar geckoes (Czarnoleski *et al*., 2017). Certainly, MRs also reflect the density and activity of mitochondria (Beaton & Hebert, 1999; Jimenez & Kinsey, 2012; Schoenfelder & Fox, 2015), and the mitochondrial activity depends on the surface area of the inner membrane (Porter, 2001) and the membrane's electrochemical potential (Hulbert, 2007) (Section VI). If small cells are energetically less demanding at certain times, mitochondrial activity can be slowed; however, small cells may achieve higher MRs, allowing fast tissue production or high physical activity.

Kozłowski *et al*. (2003*a*) modelled the effect of cell size on the mass scaling of MR. If all metabolism was dependent on the cell surface‐to‐volume ratio of cells, then the scaling of the MR at an organismal level would be 1 under a body size increase in a lineage purely *via* cell number or 0.67 under a body size increase purely *via* cell size. Because only part of metabolism is required to maintain potentials on the plasmalemma, the relationship is not expected to be so sharp, but a negative correlation should exist between the mass‐scaling exponent for cell size and the mass‐scaling exponent for MR. Indeed, Kozłowski *et al*. (2003*a*) found such a correlation in birds and mammals at the order level if the C‐value was used as a proxy for cell size (but see Isaac & Carbone, 2010).

Overall, the cellular architecture of the body should be considered when addressing the origin of MRs, but the view that ‘an animal is built of small or large cells’ may be an oversimplification. Data on cell size are still too scarce to evaluate whether cell sizes undergo concerted changes in different tissues within the body. Kozłowski *et al*. (2010) found support for such concerted changes at the interspecific level in birds and amphibians, although in mammals, not all cell types changed in complete synchrony. A comparative study of species of galliforms and rodents showed that larger species consistently evolved larger cells of five cell types (erythrocytes, enterocytes, chondrocytes, skin epithelial cells, and kidney proximal tubule cells) and smaller hepatocytes (Czarnoleski *et al*., 2018). Savage *et al*. (2007) reported diverse interspecific relationships between body mass and the size of different cell types in mammals by applying a phylogenetically non‐informed analysis. A similar inconsistency was also reported for plastic changes in cell size in response to developmental conditions (Czarnoleski *et al*., 2016, 2017).

Thus, there is little doubt that the link between cell size and MR exists and is driven by the cell‐specific MR. Cells of different size and MRs form tissues of varying metabolic activity whose proportions are one of the key mechanistic drivers of the hypoallometric scaling of MR. As we explain below, however, such an explanation of the allometry is not complete unless the problem of demand *versus* supply of oxygen and nutrients to the tissues and organs is resolved.

### (3) Demand *versus* supply

The hotly debated question of whether supply or demand is more important for determining MR and its mass scaling (e.g. Harrison, 2017, 2018*b*; Glazier, 2018*b*) is misleading because supply, demand, and constraints may be important in different scenarios (Glazier, 2018*b*). In constant environments, organisms would likely evolve simple physiology with full symmorphosis (Taylor & Weibel, 1981), although the physiology of real organisms must be complex and does not have a single universal solution. Gans (1993) and Garland & Carter (1994) criticized the idea of symmorphosis and argued that adequately matching physiological components is sufficient. Although the capacities of different components of the energy supply system show approximate harmony, a given component may be oversized at one time and constrain energy flow at another (Gebczynski & Konarzewski, 2011). Homeostatic mechanisms resulting from natural selection suffice for a limited range of environmental conditions. From the perspective of fitness maximization, the fact that some individuals in a population will die because they are unable to cope with exceptional conditions may be irrelevant; the important factor is the average success of genes and not the success of a particular individual. Thus, performance–safety compromises can be common in nature (Harrison, 2017).

**Fig 6:**
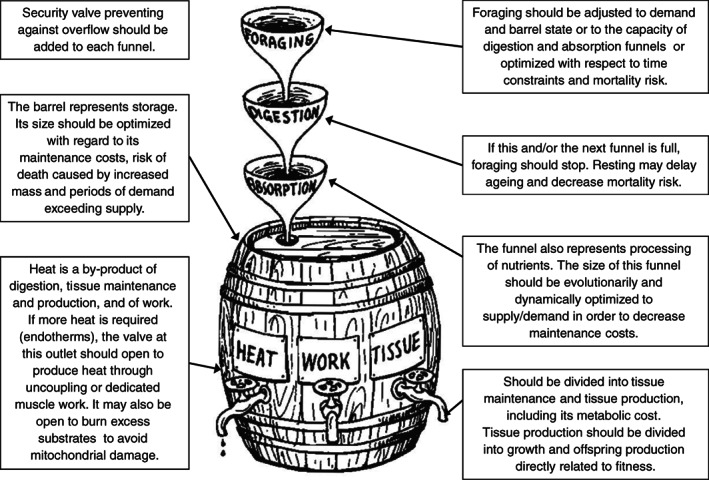
Barrel with funnels depicting energy flow in organisms; from Weiner (1992). Comments have been added to the original picture.

Maintaining homeostasis even in a limited range of environmental conditions requires numerous regulatory mechanisms (Glazier, 2015). Weiner (1992) envisioned an organism as a barrel with a cascade of input funnels and output faucets to illustrate limitations that can appear at different external or internal levels (Fig. 6). As a metaphor showing the dynamics of energy flow, this vision is overly static. The barrel has a constant volume, but the true storage volume is dynamically adjusted to address the expected imbalance in demand and supply. Funnels lack valves to protect against overflow. Funnel sizes would be evolutionarily adjusted to allow for smooth flow under constant conditions (ideal symmorphosis), although in a real and unstable world, valves must exist in the form of regulatory processes to protect against overflow. As stated by Glazier (2015, p. 3), “living things are exquisitely ‘informed resource users’”, and maintaining an organism's homeostasis requires systems that collect information about internal and external conditions of the organism and control the opening/closing of such valves.

Weiner's barrel has three output faucets: work, tissue and heat. Work requires ATP for muscle contraction. The tissue faucet includes tissue production, metabolic production costs, and tissue maintenance. Tissue production indicates somatic growth or offspring tissue/milk production. Heat is a by‐product of the flow through the ‘work’ and ‘tissue’ outlets, but in endotherms the valve at the ‘heat’ faucet opens sometimes to produce extra heat by uncoupling (oxygen consumption without ATP production) and/or muscle contraction (Section VI.1). Only the production of offspring is directly related to fitness. The other faucets and increased MR for offspring production also affect fitness, although indirectly.

Spontaneous physical activity may appear to be a waste of energy, but its role in maintaining health, which is important for escaping predators, seeking partners and preventing ageing, also in humans, is unquestionable (Levine, Eberhardt, & Jensen, 1999; Halsey, 2016). The readiness to engage in voluntary exercise varies heritably among individuals (e.g. Swallow *et al*., 2009; Brzęk *et al*., 2016). The cost of work is seemingly easy to estimate by oxygen consumption or biochemical calculations. However, such estimations represent a physiological cost rather than a fitness cost depending on the demand for heat; moreover, if all heat produced as a by‐product of work is absorbed for heating, the cost is low (Humphries & Careau, 2011) and includes only the small difference between the costs of heating *via* ATP production and uncoupling (Section VI.1). If the amount of heat produced by work exceeds the required amount, then the work may demand energetically costly active cooling. However, if the ability to dissipate heat is exceeded, it may limit energy budgets, particularly at reproduction as proposed by the Heat Dissipation Limit (HDL) hypothesis [see Speakman & Krol, 2010 for the history of this concept]. The peak sustained MR in lactating mice and bank voles may be constrained even at room temperature as evidenced by increased milk production by females with removed fur (Krol, Murphy, & Speakman, 2007; Sadowska *et al*., 2016). Nestling‐feeding blue tits (*Cyanistes caeruleus*) with removed ventral plumage sired larger nestlings, maintained lower temperature and lost less body mass (Nord, Nilsson, & Portugal, 2018). However, other studies on reproducing mammals did not support HDL predictions (Zhao, Chi, & Cao, 2010; Sadowska *et al*., 2019), thus casting doubt on its generality. Furthermore, reproducing mammals or birds do not lose fur or feathers, which is likely because of the risk of death if the temperature suddenly drops, which would on average decrease their lifetime reproductive success. Even if the HDL hypothesis works for the DEE in some circumstances as suggested by Speakman & Krol (2010), heat dissipation is unlikely to pose a general limitation on metabolic activities of tissues and organs outside of the tropics and in animals that are not very large. Furthermore, physiological or physical constraints are likely to operate on MMRs and not MinMRs; therefore, they do not account for MinMR hypoallometric scaling (Darveau *et al*., 2002). However, the level of MinMR affects the scope for activity if the HDL hypothesis works.

## VI. METABOLIC REGULATION UNDER FLUCTUATING DEMAND AND SUPPLY

### (1) Coupled *versus* uncoupled oxygen consumption

Studies on metabolic scaling customarily equate whole‐animal measures of oxygen consumption to ATP production. However, in resting rats, non‐mitochondrial oxygen consumption accounts for approximately 10% of respiration and ranges from 2 to 21% in different organs (lowest in thymocytes and highest in the liver) (Rolfe & Brown, 1997). Moreover, 15–50% of the resting oxygen consumption is attributed to so‐called proton leak (oxygen consumption uncoupled from ATP generation; a phenomenon termed ‘uncoupling’), and this proportion sharply declines with increasing MR in muscles (Melanie *et al*., 2019). Mitochondrial coupling increases with body mass in mammalian muscles, and it increases most steeply for low ATP production and least steeply for the highest ATP production (Melanie *et al*., 2019). The coupling also increases with body mass in frog livers (Roussel *et al*., 2015). In addition, equating ATP production to oxygen consumption is also inaccurate, particularly in proliferating cells, because the majority of glucose is anaerobically catabolized to lactate, which is moved to other tissues (lactate shuttle) and only later enters the tricarboxylic acid (TCA) cycle (Scott, 2005; Hui *et al*., 2017; Ferguson *et al*., 2018). As a result, the amount of ATP generated per unit of oxygen consumed (i.e. mitochondrial coupling efficiency, also termed the P/O ratio) can vary significantly (Salin *et al*., 2015). Most importantly, the P/O ratio affects such important life‐history proxies and components as the rate of growth and reproduction, the costs of somatic maintenance and lifespan (see Table 1 in Salin *et al*., 2015).

Among the mechanisms that uncouple ATP production and oxygen consumption, proton leakage has recently received particular attention, especially after the discovery of the uncoupling protein UCP1 in the brown adipose tissue (BAT) of mammals (review in Ricquier, 2017) and later in so‐called ‘beige fat’ (Schulz *et al*., 2013; Shabalina *et al*., 2013). The re‐entry of protons into the matrix through UCP1 is precisely regulated (Shabalina *et al*., 2010*b*) and is mainly a thermogenic function in mammals. The uncoupling by UCP1 is entirely reversible, and the protein is stable. The reversibility of the uncoupling performed by other UCPs, namely, UCP2 and UCP3, has not been demonstrated, and these proteins have short half‐lives ranging from 1 to 4 h in contrast to the half‐life of UCP1, which is measured in days (Azzu *et al*., 2010; Divakaruni & Brand, 2011). The amounts of UCP2 and UCP3 are so small that their roles in oxygen consumption are probably negligible (Shabalina *et al*., 2010*a*). The functions of UCP2 and UCP3 are still unknown, but these proteins seem to have roles in defence against free radicals (Shabalina *et al*., 2013).

Although the function of UCP1 in BAT seems indisputable, it cannot account for the ubiquity of uncoupling because brown fat cells are absent in birds and scant in large mammals, including adult humans (Rowland, Bal, & Periasamy, 2015). Additionally, the functions of orthologues of UCP1 that occur even in ectotherms are unclear (Hughes *et al*., 2009). However, another mechanism accounts for 1/2 to 2/3 of basal proton conductance as revealed when both ATP production and induced uncoupling through specialized proteins are blocked (Brand *et al*., 2005). This mechanism can be attributed to the abundance, but not the activity, of adenine nucleotide translocase (ANT) (Divakaruni & Brand, 2011) and to the electrochemical potential across the inner mitochondrial membrane (Liesa & Shirihai, 2013). However, neither UCPs nor ANT can fully explain the observed levels of uncoupling, which must therefore be attributed to other mechanisms. Emerging studies suggest the existence of a potentially important uncoupling process outside the mitochondria: futile sarcoplasmic reticulum calcium ATPase (SERCA) pump activity (Pant, Bal, & Periasamy, 2016). The sarcolipin‐mediated uncoupling of SERCA may therefore serve as a potential mechanism for thermogenesis in animals that lack BAT or beige fat.

Aside from the obvious thermogenic aspect, other roles of induced uncoupling have been suggested and appear to be important not only for endotherms but also for the high absolute aerobic scope of muscles in ectotherms (Clarke & Portner, 2010). The same cells must change their ATP production flexibly to satisfy the balance of supply and demand. Long‐term (e.g. seasonal) differences in demand can be satisfied by changing the mitochondrial density in cells. Middle‐term (hours) changes in demand can be satisfied by the fusion/fission or tethering/untethering of mitochondria, where long mitochondria or chain formation promotes oxidative phosphorylation, whereas fragmentation accompanies an uncoupled state of mitochondria (Liesa & Shirihai, 2013; Toyama *et al*., 2016), such as in hormone‐induced non‐shivering thermogenesis (Wikstrom *et al*., 2014). However, short‐term changes in demand (minutes and seconds) also occur, especially in the muscles. When there is high demand for ATP in a working tissue, the delivery of substrates is accordingly adjusted to be balanced with its utilization. Slowing this delivery after a sudden drop in ATP demand *via* pancreatic beta cell signalling for proper insulin production requires time. Meanwhile, a temporary excess of substrates could appear in the mitochondria, which has devastating consequences for mitochondrial health, including an increase in membrane potential and therefore in reactive oxygen species (ROS) production (Liesa & Shirihai, 2013) because ROS production increases with membrane potential (Brand, 2000; Brand & Esteves, 2005; Barja, 2014). To avoid this dangerous state, the membrane potential can be decreased by the induced re‐entry of protons to the matrix, which allows the excess of substrates to be burned away (Liesa & Shirihai, 2013). If the main role of induced uncoupling, apart from thermogenesis, is to burn excess substrates, then the induced proton leakage should be downregulated under high ATP demand. According to Rolfe *et al*. (1999), uncoupling dropped from 52 to 34% when muscles were at work and the proton leak was responsible for only 22% of the oxygen consumption in the working liver compared to 26% in this organ at rest. The induced proton leak after hard muscle work may be a component of so‐called excess post‐exercise oxygen consumption, a phenomenon studied in sports medicine (e.g. Schleppenbach *et al*., 2017) but rarely in physiological ecology (Fu *et al*., 2009; but see e.g. Hancock & Gleeson, 2002; Zhang *et al*., 2014).

Undoubtedly, ROS production is not a simple derivative of the total MR as measured by oxygen consumption. Stier *et al*. (2014) exposed wild‐type and UCP1‐deficient mice to moderate cold for 4 weeks and found that the animals in both groups had the same metabolic level, which was achieved by wild‐type mice *via* non‐shivering thermogenesis and by UCP1‐deficient mice *via* shivering thermogenesis. Increased oxidative stress was noted only in the latter group, which means that uncoupled oxygen utilization does not significantly increase ROS production, whereas the production of ATP does. Zebra finches (*Taeniopygia guttata*) treated with the artificial mitochondrial uncoupler 2,4‐dinitrophenol (DNP) had elevated MRs but were able to maintain the same body mass as controls due to increased food consumption, and elevated MR did not cause increased oxidative stress (Stier *et al*., 2014). The zebra finches treated with DNP had less DNA oxidative damage than the controls when exposed to acute (but not chronic) cold. Mice uncoupled with DNP were smaller, had lower oxidative stress and lived longer (Caldeira da Silva *et al*., 2008). Unlike zebra finches, mice were unable to compensate for an elevated MR with increased consumption; thus, their ATP production was lower. Tadpoles of the frog *Rana temporaria* treated with DNP consumed the same amount of food as control frogs, produced less ATP and had less oxidative damage despite lower antioxidant production (Salin *et al*., 2012). All these experiments suggest that only the part of the metabolism related to ATP production is correlated with ROS production. The roles of ATP production and uncoupling in oxygen consumption should be considered in hypotheses on ageing related to mitochondrial deterioration. The cited results favour the ‘uncoupling to survive’ hypothesis (Brand, 2000) and not ‘the rate of living – free‐radical damage’ theory (Pearl, 1928; Harman, 1956; Sohal, 2002). Speakman *et al*. (2004*b*) found a positive correlation between oxygen consumption and longevity in mice, and animals with oxygen consumption in the upper quartile were characterized by higher proton leak.

The role of uncoupling in the prevention of excess ROS production is still controversial (e.g. Shabalina & Nedergaard, 2011). Assuming a direct relationship between uncoupling and ROS production may be one underlying reason. If uncoupling is a way to restore substrate balance after a drop in ATP demand, the frequency and magnitude of fluctuations in demand should be considered along with the importance of keeping muscles in readiness for increased aerobic effort. The cost of uncoupling may be negligible in endotherms below the thermoneutral zone because waste heat can be used to keep the animal warm; on the other hand, the cost may be very high above the thermoneutral zone because costly additional cooling is necessary. Thus, we can expect lower uncoupling in warm conditions, which is measured as lower BMR, accompanied by an impaired ability to switch rapidly to a high aerobic metabolism. An important point to consider is that the deterioration of mitochondria is not a tragedy as long as healthy mitochondria can be selected and can proliferate (Section VI.3).

This brief review of coupling/uncoupling mechanisms demonstrates that following absorption, oxygen atoms often enter different molecular pathways that cannot be equated to ATP production. Consequently, mitochondrial coupling efficiency may vary considerably (Salin *et al*., 2015) and cannot be ignored in studies on metabolic scaling. This is of particular importance for a better understanding of the physiological and molecular mechanisms underlying the slow–fast/frugal–wasteful continuum (Hou & Amunugama, 2015), which is essential to our reasoning.

### (2) Cell membrane composition and MR

The idea that the hypoallometric mass scaling of MR relates to the fatty acid composition of membranes was introduced by Hulbert & Else (1999). Indeed, the percentage of the omega‐3 unsaturated fatty acid docosahexaenoic acid (DHA) decreases with increasing body mass in birds and mammals (Hulbert, 2007). The omega‐3/omega‐6 ratio tends to decrease with increasing body mass in the skeletal muscles of mammals (Hulbert, Rana, & Couture, 2002b) and birds (Hulbert *et al*., 2002*a*) and in the hearts of mammals (mouse, rat, sheep, and cow, but not pig) but not birds (zebra finch, house sparrow *Passer domesticus*, starling *Sturnus vulgaris*, currawong *Strepera graculina*, pigeon *Columba livia*, mallard *Anas platyrhynchos*, graylag goose *Anser anser*, and emu *Dromaius novaehollandiae*) (Turner *et al*., 2006). The amount of omega‐3 polyunsaturated fatty acids (PUFAs) in the liver, kidneys and brain of mammals either was independent of body mass or decreased only slightly with increasing body mass (Hulbert *et al*., 2002*b*). Changes in the fatty acid composition of cells with body mass seem to depend on tissue type and on taxonomic position to some extent. Brookes, Hulbert, & Brand (1997) did not find a relationship between membrane composition and proton leak in liposomes (structures without membrane proteins) and proposed a relationship between fatty acids and the activity of membrane proteins. Turner *et al*. (2006) also suggested a potential association between membrane lipid composition and the activity of membrane‐bound Na^+^, K^+^‐ATPase in the hearts of endotherms. According to this line of reasoning, we suggest that fatty acid composition does not directly affect metabolic processes apart from their possible signalling role. Because particular types of PUFAs may be related to particular transmembrane proteins, a change in their abundance would follow changes in the amount of transmembrane proteins. PUFAs are speculated to provide elasticity for transmembrane enzymes, thus affecting their work (Andersen & Koeppe, 2007; Bruno, Koeppe, & Andersen, 2007). Poveda *et al*. (2014) suggested the segregation of particular PUFAs to specific ion channels to ensure a defined milieu around the protein differing from the bulk membrane composition, which is called an annular lipid shell (Contreras *et al*., 2011) or a space‐filling sealant (Valentine & Valentine, 2010). A bilayer thickness close to that of transmembrane proteins is particularly important because a hydrophobic mismatch between fatty acids and proteins causes membrane deformation, resulting in improper functioning of the protein gates (Andersen & Koeppe, 2007; Mondal, Weinstein, & Khelashvili, 2012).

The link between bilayer composition and MR seems mostly indirect, which explains the ambiguous results described in the literature. Therefore, determining the physio‐chemical relationships between bilayer composition and membrane proteins may be more fruitful than identifying correlations between the abundance of fatty acids, body mass and MR.

### (3) Cells never sleep

The question of hypoallometric scaling of MR, particularly of MinMR, is in its essence the question of scaling of the energetic costs of maintenance of cells (Fig. 3A). Resting cells allocate an important fraction of energy for repair processes, partly through autophagy and the ubiquitin‐proteasome system, which are processes that degrade and recycle damaged or unnecessary intracellular components. These processes directly affect the rate of ageing and are controlled by the nutrient‐sensing protein kinase complex of mTOR and TORC1 (Rousseau & Bertolotti, 2016).

The prominence of autophagy and proteasome activity indicates that distinguishing between ‘structure’ and ‘reserve’ (Kooijman, 2010) is biologically questionable. What is ‘structure’ in one moment may become ‘reserve’ in another, not only when damaged but also when no longer necessary or simply not indispensable under starvation stress. We should not equate living things to a car with a tank (structure) filled with fuel (reserve). Organisms are more similar to a house in winter with a fireplace and its inhabitants: with a smooth resource supply, heat is produced from the reserve wood, but when the wood supply does not cover the demand, the inhabitants start to burn unnecessary furniture, and when supply exceeds demand, wood can be used to rebuild the items that have been burned.

There is a special form of autophagy called mitophagy that degrades mitochondria that are damaged or in excess. Mitochondria tend to fuse and form long structures when the demand for ATP is high. When the demand drops and uncoupling intensifies, mitochondrial fission prevails (Liesa & Shirihai, 2013). This fusion/fission process forms an open cycle because its purpose is twofold: adjusting the ATP supply to the current demand and performing quality control. Damaged mitochondria are destroyed. If too many mitochondria are damaged, a signal for cell apoptosis is produced. The biochemical mechanism of fission mediated by AMP‐activated protein kinase was recently described by Toyama *et al*. (2016). Mitophagy may also remove excess healthy mitochondria after a chronic decrease in ATP demand, such as a seasonal decrease. The fragmentation of mitochondria is also important for the proliferation of healthy mitochondria if the demand for ATP is chronically increased. The chronic overfeeding of mitochondria may disturb their fusion/fission cycles and thus their selection, which results in their gradual deterioration and ageing (Liesa & Shirihai, 2013). Conversely, the decreased rate of ageing that follows a restricted diet results at least partly from enhanced autophagy and proteasome activity, which keeps cells and mitochondria healthy for a longer time, thus diminishing the necessity for cell replacement and preserving the pool of stem cells (Gelino *et al*., 2016). When nutrients are available in excess at a cellular and especially mitochondrial level, ATP production becomes less efficient, and nutrient oxidation is more strongly directed towards heat production through uncoupling in order to restore balance (Liesa & Shirihai, 2013).

Importantly, the inherent dynamics underlying the balance of nutrients and mitochondrial oxidation most likely also account for the less‐than‐perfect correlation between MinMR and ageing after controlling for body mass (see examples in Selman *et al*., 2008; Speakman, 2005; Speakman *et al*., 2004*b*). This correlation is further eroded by differences in energetic costs of repair mechanisms, because some of them are ATP dependent [e.g., base excision repair (BER) system (Maher *et al*., 2017)], while others (such as dismutases) are ATP independent (Rulisek *et al*., 2006). Differences in energetic costs of repair may also explain why larger animals that are built with a larger number of cells and have a lower cell‐specific MR are no more susceptible than smaller animals to damage‐related malignancies, such as cancer, which is called Peto's paradox (Peto *et al*., 1975). The very existence of this paradox suggests that the cells of larger animals tend to be less prone to damage or are more effectively repaired or replaced.

The question of why autophagy, proteasome activity and other repair mechanisms are not kept at the optimal level from the perspective of cell health when food is abundant has a simple answer: neither cell health nor an organism's longevity but the expected lifetime offspring production, which considers ecological mortality, is the target for natural selection. Medicine, which is not constrained by Darwinian fitness maximization, can extend longevity beyond the horizon dictated by natural selection.

## VII. RECOMMENDATIONS FOR FUTURE RESEARCH

**Fig 7:**
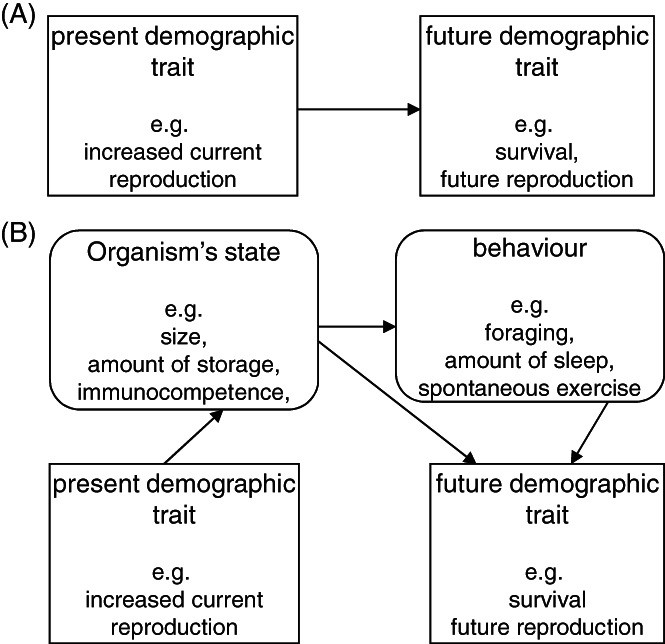
Two approaches to life‐history evolution: (A) purely demographic changes, such as when a change in one demographic trait alters another demographic trait; and (B) changes in one demographic trait alter the organism's state and the new state determines another demographic trait.

There are two approaches in the studies of life‐history evolution (Fig. 7). The first approach is purely demographic and it assumes that a change in one demographic parameter affects another demographic parameter (Fig. 7A). Thus, a trade‐off is assumed between demographic parameters; for example, increased reproduction impairs either future reproduction or survival. Such an approach allows for comparisons of the average values for species, populations, or subsets of a population that represent a uniform strategy, whereas differences between individuals must be ignored. This approach is exemplified by numerous studies (e.g. Abrams, 1991; McGraw & Caswell, 1996). The second approach, which we advocate herein, highlights the role of the organism's state (Fig. 7B). Alteration of one demographic parameter changes the organism's state, the state impacts the organism's behaviour, and the state and behaviour alter other demographic parameters (future reproduction or survival). For example, increasing current reproduction may drain resources from growth and thus lower future reproduction if fecundity is size dependent (e.g. Reznick, 1983; Lester *et al*., 2004). Another potential strategy is to save on the immune system to increase fertility at the cost of survivability or future reproduction (e.g. French, DeNardo, & Moore, 2007; Knowles, Nakagawa, & Sheldon, 2009). Engaging in risky foraging to maintain growth rate at the cost of survival is another potential strategy (Mathot *et al*., 2019). The approach represented in Fig. 7B allows for the incorporation of individual differences in physiology or personality.

Irrespective of the adoption of one of the approaches outlined above, it is always necessary to distinguish between the two continua of life strategies discussed in Section III: strategies governed by the efficiency of production, i.e. the wasteful–frugal continuum, and strategies governed by ecological mortality. We cannot understand covariation between life history, including the rate of senescence, and physiology when we mix these two axes. The existence of the two life rate axes also warns against automatically eliminating body mass effects in studies of organisms because a position along each axis is strongly linked to adaptive body mass (Fig. 4). We must forget about treating body mass as an independent variable and consider it very carefully before we decide to mass‐standardize any organismal trait. For example, if high mortality is an evolutionary driver of small size, then standardizing mortality rate to body mass in studying the relationship between mortality rate and age at maturity is akin to throwing the baby out with the bathwater. Furthermore, if we accept that scaling equations are only statistical tools to describe the relationship between body mass and traits, papers presenting such equations are worthy of publication if such equations support or falsify some specific hypotheses other than considerations of whether the scaling exponent is closer to 3/4 or 2/3. Moreover, papers devoted to pure comparisons of scaling exponents between taxa without verification of specific hypotheses will also not push science forward very much.

Furthermore, understanding the coevolution of body size and MR requires knowledge of within‐species scaling of the adult production rate (Section II). Focus should be changed from studying the scaling of whole‐body MR to studying the scaling of the production rate under natural conditions, with an underlying role of MR. It is important to find out whether production rate shows hypoallometric scaling with body mass as predicted in Section II. Because measuring production rates is much more difficult than measuring MR, it is also important to establish whether assumptions on the proportionality of production rates and MRs are at least roughly justified.

Integration of demographic and physiological approaches within the wasteful–frugal and ecological mortality continua seems indispensable for a better understanding of the coevolution of MR and body size. For physiologically oriented researchers, it is enough to understand when intrinsic population growth rate and when lifetime offspring production is a proper measure of fitness (Section II; Dańko *et al*., 2018), which does not require a full understanding of mathematical models. They must also properly understand what the term ‘lifetime offspring production’ means, especially how it is related to mortality (Section II). The same mechanisms, which have a genetic basis, are shared by many individuals. It does not matter if imperfect physiology causes the death of many if, on average, it provides the highest lifetime reproductive success thanks to lucky survivors. Because expected‐at‐birth lifetime offspring production depends not only on productivity but also on survivability, the ecological mortality rate must affect not only body size but also metabolic patterns (Section II). We suggest that ecological physiologists should practice seeing in each individual a representative of a given strategy; therefore, how such strategies affect Darwinian fitness rather than the owner's fitness (fitness in the common sense) is important. Only medical or veterinary doctors should be interested in individual well‐being, although even these doctors can gain from such an integrated field: they can learn what can be improved in our imperfect physiology that evolved not for individual well‐being but for Darwinian fitness maximization, when we or our domesticated animals no longer live under the Darwinian fitness maximization dictatorship.

Understanding the coevolution of MR and body size should encourage the elimination of single‐cause explanations of the hypoallometric scaling of MR and lead us to address the question of why the scaling is hypoallometric. This question directs us towards studying MR at the tissue/cell level (Section V). Here, the recent paradigm shift must be considered because glycolysis is no longer viewed exclusively as a rescue strategy under oxygen deficit but also as the major biochemical process required for proliferating cells because of stoichiometric requirements (Vander Heiden, Cantley, & Thompson, 2009; Hui *et al*., 2017). Lactate produced as a by‐product becomes a fuel for tissues with high ATP demand, such as muscles, liver or brain (Hui *et al*., 2017) during the lactate shuttle (Brooks, 2018) or can be recycled back to glucose in the energetically expensive Cori cycle and/or to triglycerides as precursors of fat. Understanding that physiological processes performed by a given tissue are not isolated from each other becomes especially important in studying the metabolism of organisms with intense proliferation of cells, for example, organisms growing in size. Lactate metabolism, which so far has not been embraced by the majority of eco‐physiologists, should become one of the key directions for future research on MRs.

Although the shift toward studying metabolic processes at the cellular level seems unavoidable, it is necessary to remember that cellular metabolism is regulated at the organismal level (Darveau *et al*., 2002; Suarez & Darveau, 2005; Glazier, 2014, 2015). Thus, studying MR at the level of individuals under different environmental and behavioural circumstances makes sense, but must be accompanied by reflection on how such crude measures depend on the ongoing processes in tissues/cells. In MR studies, organisms cannot be treated any longer as black boxes. It is especially important to distinguish between such tissues as muscles, with their low maintenance costs at rest and extremely high ATP demands at work, and the brain, which has a constantly high energy requirement. It also seems necessary to distinguish, at least conceptually, between ATP production and uncoupling if the MR is measured by oxygen consumption or CO_2_ production (Section VI.1; Salin *et al*., 2015). In addition to the obvious thermogenic effects of uncoupling, its role in burning excess substrates after a sudden drop in demand for ATP to prevent mitochondrial damage requires further investigation. Without distinguishing between ATP production and uncoupling, unravelling the relationship between MR, ROS production and ageing may be impossible because uncoupling does not increase ROS production and may even prevent its production (Section VI.1). As highlighted in Section VI.1, an in‐depth understanding of those molecular pathways will be indispensable for the identification of mechanisms underlying the significance of the slow–fast/frugal–wasteful continuum. We also urge for studying causal connections between fatty acids and transmembrane proteins instead of correlating membrane composition with MR or mitochondria/cell health (Section VI.2).

The role of cellular architecture in the evolution of body size and MR requires more attention. Since research on cell size is extremely laborious and never complete because there are too many tissues and organs to be studied, it is necessary to establish whether the amount of DNA (C‐value) can be used as a proxy for an average cell size at the interspecific level. If the answer is ‘yes’, which is likely (Section V.2), then studying the C‐value together with body mass along phylogenetic trees may help to clarify the coevolution of body mass and MR and to solve the so‐called C‐value enigma.

The static view of structures and processes contributing to MRs must be abandoned. Not only are cells replaced but so also are structures within cells. The dynamics of mitochondria are of particular significance because they undergo fusion/fission process, with fusion prevailing at high ATP demand and fission promoting uncoupling (Section VI.3). Open fusion/fission cycles also eliminate damaged mitochondria (mitophagy) and serve to multiply healthy ones. Further studies are required to understand the relation between mitochondrial dynamics and the metabolic states of cells and organisms. Importantly, autophagy, including mitophagy, is important for slowing down the ageing process, but these processes require undernutrition of cells. Thus, conditions that promote autophagy, such as a periodic low food supply to cells, must be present to extend life. Energetically cheap or cost‐free repair mechanisms involved in the relation with autophagy may not compete with growth and reproduction, as illustrated in Fig. 3A, but instead may extort lower resource acquisition, with the same effect as direct drainage of energy: slower growth and less‐intense reproduction. We suggest that adopting such an approach may be the way forward to resolve many controversies related to the elusive metabolic costs of reproduction, the lack of a straightforward relationship between ROS production and MR and the positive effect of restricted diet on life extension.

## VIII. CONCLUSIONS

(1) The overwhelming wealth of physiological, behavioural, ecological and evolutionary processes that affect MR leads us to conclude that the quest for one universal mechanism explaning the mass scaling of MR is futile. There is a central tendency for MR to increase at a slower than linear rate with body mass (hypoallometric scaling), although because of the wide scattering of data points around the approximation line, it is possible to find small species with higher MRs than those of larger species. In our opinion, scaling equations do not represent any deep biological laws but rather are statistical descriptions of the relationship between two variables and an approximation of non‐linearity, which is not always perfect because more and more cases of non‐linearity on the log–log scale relationship are being discovered. Accepting such a view will free science from the overflow of papers representing one‐cause mechanistic explanations of hypoallometric scaling.

(2) What determines the hypoallometric scaling of MR, especially at a low basal or standard metabolic level? The proximate mechanism consists of relatively smaller energy‐demanding visceral organs, especially the brain, and their lower mass‐specific MR in large species (Section V.1). Larger‐on‐average cells in larger species are likely to contribute to the decrease in mass‐specific MR of visceral organs with body mass. Ultimate factors must be considered from a life‐history perspective involving the coevolution of MR, production rate *P*(*w*) and body mass (*w*) under the selective pressure of ecological mortality *m*(*w*). Evolved body size must be placed in the region for which *P*(*w*)/*m*(*w*) and usually also *P*(*w*) are concave downwards (Section II). If *P* is on average roughly proportional to MinMR, then this condition provides an ultimate explanation of the hypoallometric scaling of MR at the intraspecific level, which is translated to the interspecific level through the coevolution between body mass and MR (Section IV.3). Large animals have much lower mass‐specific MinMR and lower mass‐specific MMR not because they are constrained, but because they do not need higher MRs to pass on their genes most effectively.

(3) From the perspective of proximate factors, seeking limitations to metabolism that cause hypoallometric scaling seems to be a natural approach. However, the ultimate factors are those that maximize fitness, which is usually the lifetime offspring production. We agree with Harrison (2018*a*) that different limiting factors for MR described in the literature may be constraints only from a physiological point of view because compensating mechanisms are likely to evolve. For problems with heat dissipation, special appendages can be evolved for cooling or hair/feathers can be lost. For difficulties with supplying oxygen, pneumatic bones and air sacs evolved in dinosaurs more than 200 million years ago, long before birds originated (Brusatte, 2017), as a response to the low oxygen level in the atmosphere in the late Triassic (Ward & Kirschvink, 2015). For insufficient blood for delivering nutrients and oxygen to heavy‐working muscles, the capillary bed could be denser, and its density in fact increases after endurance training both in humans and other mammals (Egginton, 2009). Many constraints exist because overcoming them would decrease fitness and not because overcoming them is impossible. As stated in Section V.3, organisms should be only quasi‐symmorphic and not ideally symmorphic; thus, different limitations on MR are unavoidable.

(4) There is a common tendency for the human mind to believe that similar results have similar causes; however, this reasoning is misleading both in everyday life and in science. Such false reasoning has led 100s of researchers to search for a universal explanation of the specific parameters of the hypoallometric scaling of MR. Adoption of the life‐history perspective on physiological ecology advocated here leads to the conclusion that no such explanation exists. Instead, this perspective refocuses studies of the scaling of MR appropriately on the mechanisms of natural selection and the maximization of Darwinian fitness.

## Supporting information


**Appendix S1** Primer on scaling and curve shapes with arguments suggesting why these issues are important for the study of body size variation.

**Appendix S2** Intraspecific evolution of body mass affects interspecific mass scaling of metabolic rate.
