# Supplementary material for: Coevolution of body size and metabolic rate in vertebrates: a life‐history perspective
Source: Biol Rev Camb Philos Soc. 2020 Jun 10;95(5):1393–417. doi: 10.1111/brv.12615 (PMC7540708; doi:10.1111/brv.12615)
Supplement: Supplementary file 1 — Appendix S1 Primer on scaling and curve shapes with arguments suggesting why these issues are important for the study of body size variation. [file BRV-95-1393-s001.docx]

**Appendix S1. Primer on scaling and curve shapes with arguments suggesting why these issues are important for the study of body size evolution**

**Scaling**

Recently, the term ‘scaling’ has been used more frequently than ‘allometry’. Scaling is usually modelled by a simple allometric function *Y* = *aX^b^*, and its logarithmic transformation log *Y* = log *a* + *b*log *X* is most often used for practical reasons (Fig. S1). Logarithmic transformation should be used not only for linearization but also for normalization of the distribution of data points around the regression line (Glazier, 2013). The scaling exponent *b*, or the slope in the logarithmic form of the equation, is equal to 1 for isometry, i.e. *Y* increases linearly with increases in *X* (solid red line in Fig. S1A). If *Y* increases with *X* at a faster than linear rate, then *b*>1 and the allometry is positive or hyperallometric (dashed green line in Fig. S1A). If *Y* increases with *X* at a slower than linear rate, then *b*<1 and the allometry is negative or hypoallometric (broken black line in Fig. S1A). Note that in the logarithmic form, all three lines are straight and the slopes are equal to 1, greater than 1 or less than 1 (Fig. S1B). We use the term ‘hypoallometric’ for negative allometry (*b*<1) in our paper. For a decreasing allometric function, the exponent *b* is negative. The metabolic rate usually increases with body mass at a hypoallometric rate in interspecific comparisons, although it can have more complex non-allometric forms over the course of ontogeny (Glazier, 2005).


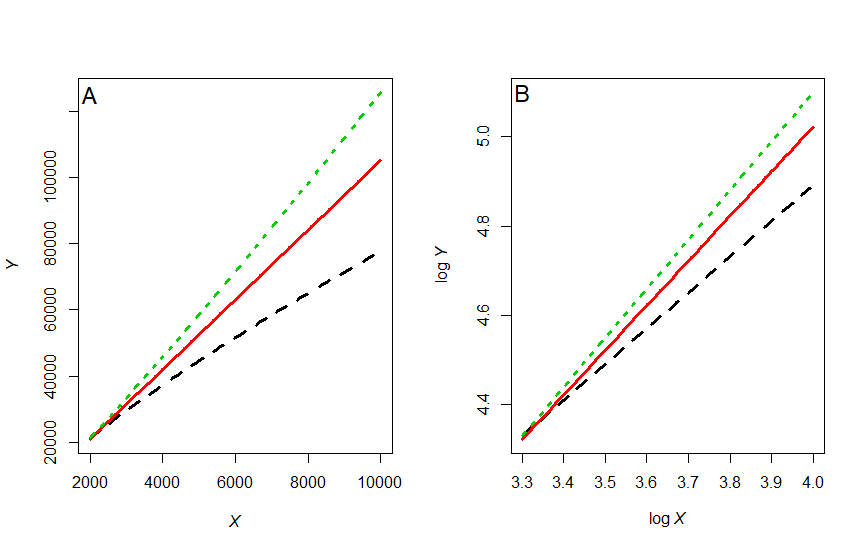


**Fig. S1.** Relationship between two traits (*X* and *Y;* arbitrary units) described using a power (allometric) function *Y* = *aX^b^*, which is shown by normal (A) or logarithmic (B) coordinates. Red solid line indicates isometry (*b* = 1); broken black line indicates negative allometry/hypoallometric scaling (*b*<1); and dashed green line indicates positive allometry/hyperallometric scaling (*b*>1).

**Curvature of functions**

The allometric function with an exponent 0<*b*<1 is a special case of a monotonically increasing concave function. Because the terms ‘concave’ and ‘convex’ are often confused by non-mathematicians, we use the synonym ‘concave downwards’ for the concave function (Fig. S2, dashed black line) and ‘concave upward’ for the convex function (Fig. S2, red broken line). A monotonic increase means that the function never decreases and has no local maximum and an always positive first derivative (tangent line to the function line). More complex functions of *Y* can have concave downwards regions within some range of *X* and concave upward regions in other ranges of *X* (Fig. S2, green solid line). The point where such functions change in curvature is called an ‘inflection point’. The second derivative of the function is equal to zero (necessary condition) and changes its sign (sufficient condition) at the inflection point.


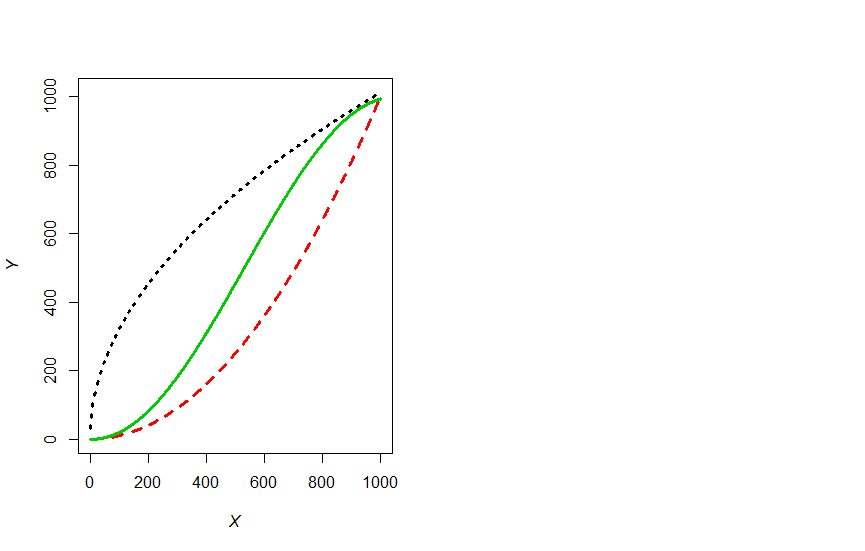


**Fig. S2.** Basic shapes of monotonically increasing functions of *Y* *versus* *X*: red broken line represents the convex function (concave upward); black dashed line represents the concave function (concave downwards); and green solid line represents a complex function with an inflection point that divides the function between its concave downwards and concave upward parts. More than one inflection point can occur in a function and functions may not increase monotonically.

**Scaling and curvatures facilitate an understanding of the evolution of body size**

The shape of a production rate/mortality rate function, *f*(*w*) = *P*(*w*)/*m*(*w*) where *w* represents body size, is important for optimal (adaptive) body size of adults and their offspring. If this function is concave downwards, an optimal adult size exists, but an offspring size is then as small as possible, because it is better to produce more small offspring unless some constraint prevents further decrease in offspring size. If *f*(*w*) is concave upward, an optimal offspring size exists, but adult size should be as large as possible, unless limited by some constraint. The existence of an inflection point along the function *f*(*w*) is a necessary condition for the existence of optimal sizes for both offspring and adults (Kozłowski, 1996*c*).

It is optimal to allocate surplus resources not used for maintenance to either growth or reproduction but not to both processes simultaneously. Such an instantaneous switch may be reversible in animals with indeterminate growth, such as fish, or irreversible in animals with determinate growth (Kozłowski, 2006). In the latter, the function *f*(*w*) measures how many energy units an animal can expect to allocate to offspring if it ‘decides’ to stop growing and mature at a given body size *w* and then devote all available resources not used for maintenance to reproduction. This is because *P*(*w*), in units such as J/day, measures the rate of production per day, and 1/*m*(*w*) measures life expectancy in days. If body size is measured in energy units and efficiencies of building the animal’s own tissues and offspring tissues are the same, then the adult optimal size is that for which the first derivative of *f*(*w*) is equal to 1. The derivative is greater than 1 below the optimum and smaller than 1 above the optimum. In other words, each Joule of energy allocated to growth will increase the expected offspring production by more than 1 J below the optimal adult size (it is adaptive to continue growing), and less than 1 J above the optimal adult size (it is adaptive to reproduce). If a concave upward region of *f*(*w*) occurs, the optimal size of an offspring *w*_0_ is that for which *w*_0_*m*(*w*_0_) = *P*(*w*_0_). The left-hand side of this equation represents biomass loss through mortality, and the right-hand side represents biomass gain through the production capacity of tissues. Above *w*_0_, the gains are larger than losses, and because of the concave upward *f*(*w*), an animal will grow at an increasing speed (Kozłowski, 1996*c*; Taylor & Williams, 1984). If body size and offspring production are measured in different units or production rate differs depending on the sink of allocation, the equations are more complex but the problem is still tractable (Kozłowski & Wiegert, 1987). Adding more biological variables may result in analytically non-tractable solutions, although in this case, numerical methods, mainly dynamic optimization, can be used to find optimal sizes.
