# Supplementary material for: Coevolution of body size and metabolic rate in vertebrates: a life‐history perspective
Source: Biol Rev Camb Philos Soc. 2020 Jun 10;95(5):1393–417. doi: 10.1111/brv.12615 (PMC7540708; doi:10.1111/brv.12615)
Supplement: Supplementary file 2 — Appendix S2 Intraspecific evolution of body mass affects interspecific mass scaling of metabolic rate. [file BRV-95-1393-s002.docx]

## **Appendix S2. Intraspecific evolution of body mass affects interspecific mass scaling of metabolic rate**

Kozłowski & Weiner (1997) simulated the origin of the natural diversity of body sizes among species. They allowed imaginary species to mature at body masses that maximize fitness according to the model of optimal resource allocation discussed in Section II of our paper. Each simulated species ‘evolved’ under a range of selective conditions, including a unique combination of parameters that defined the species-specific mass dependence of the mortality rate, resource acquisition (assimilation) and metabolic rate (MR). Most importantly, Kozłowski & Weiner (1997) did not assume one universal mass scaling of metabolism for all species. Rather, the species-specific exponents ranged from 0.57 to 0.86, and were randomly selected from a normal distribution with 0.75 as the mean value. Similarly, other parameters for defining resource acquisition and mortality were also selected at random (for a full description of the model see Kozłowski & Weiner, 1997).

Next, each species was described by its evolved adult body mass and the resulting MR, and these data were then used to calculate an interspecific mass scaling of the MR. This procedure resembles a real situation in which species evolve their adult sizes in specific environments in response to selection pressures. A researcher interested in metabolic scaling measures body mass and MR of individuals of a studied species, then fits a regression line to a log–log plot to estimate the mass-scaling exponent (a slope of this regression).

Kozłowski & Weiner (1997) found that despite the apparent high variance in the species-specific exponent values, the data on MR *versus* adult body mass at the interspecific level created the illusion that all species were characterized by a common mass scaling of metabolism (Fig. S3). Furthermore, the interspecific value of the exponent (0.70) was lower than the mean exponent value (0.75) in the distribution used as the source of sampled species-specific exponents. The stabilizing effect of body-size optimization on interspecific allometries and their scaling is explained in Fig. 5 in the main text. The mortality rate had no effect on the interspecific slope but was the most influential determinant of the body mass distribution of the simulated taxon. Interestingly, the simulations produced more small species than large species, which is understandable because low mortality and high production are needed for a large size to be optimal while a small (but not extremely small) size may be optimal under conditions of low production or high mortality, either together or separately. The interspecific dependence of the production rate becomes almost linear (Fig. S3), although it is non-linear in most species.

An important insight is that body mass is not an independent variable for MR in an evolutionary sense as opposed to a purely statistical sense, because differences in the mass scaling of metabolism among species drive the evolution of adult body mass within these species, which in turn causes a difference between the interspecific and intraspecific mass scaling of MRs.


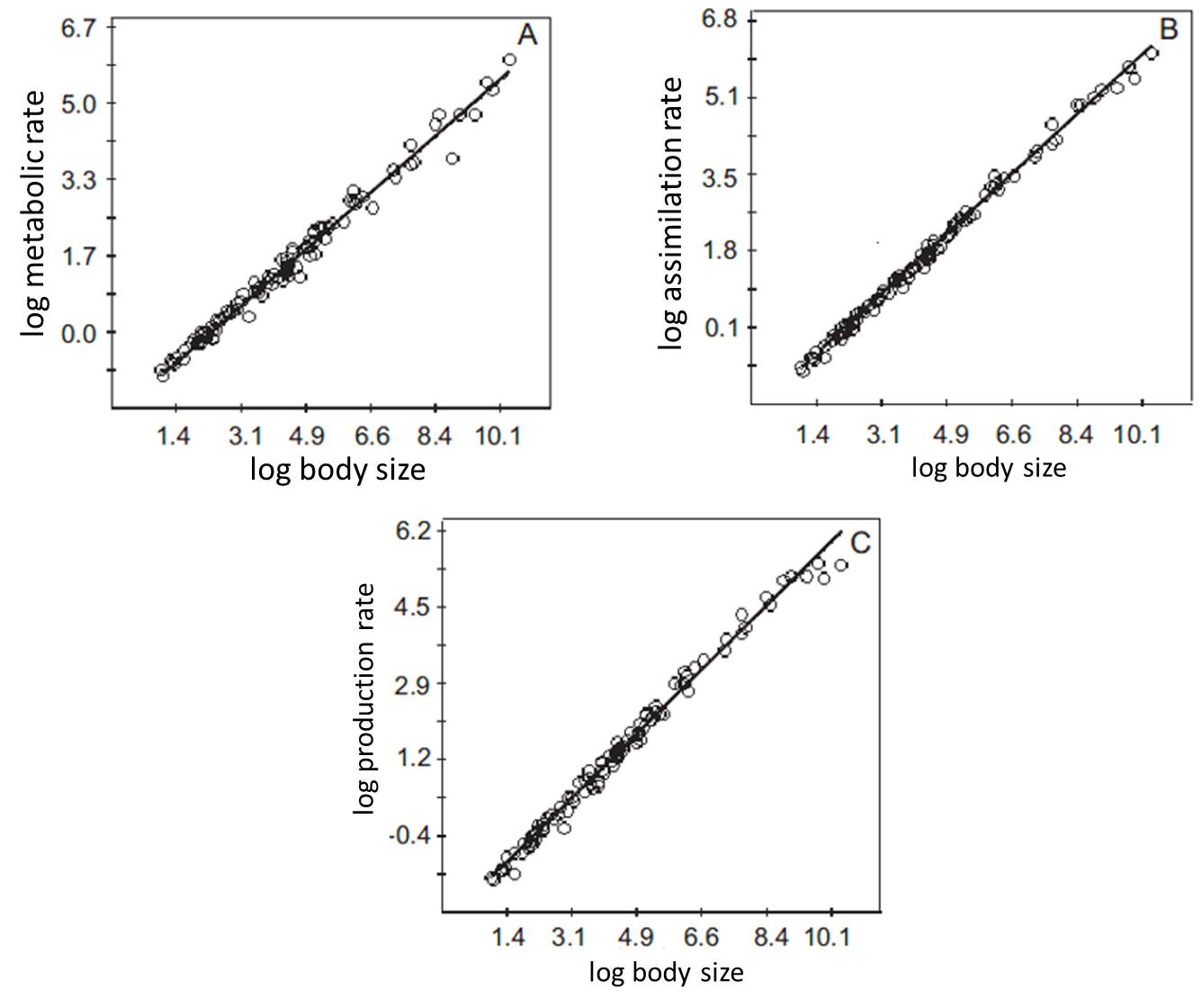


**Fig. S3.** Interspecific scaling of (A) metabolic rate (MR), (B) assimilation rate, and (C) production rate (i.e. the difference between assimilation and MR) of 100 simulated species, with randomly selected species-specific exponents for mass scaling of the rates (see appendix text for explanantion). After Kozłowski & Weiner (1997).
